# Observing cognitive processes in time through functional MRI model comparison

**DOI:** 10.1002/hbm.26114

**Published:** 2022-10-26

**Authors:** Michael Marxen, Johanna E. Graff, Philipp Riedel, Michael N. Smolka

**Affiliations:** ^1^ Department of Psychiatry Technische Universität Dresden Dresden Germany

**Keywords:** amygdala, attention capture, emotions, fMRI, insula, model comparison, neuroimaging

## Abstract

The temporal specificity of functional magnetic resonance imaging (fMRI) is limited by a sluggish and locally variable hemodynamic response trailing the neural activity by seconds. Here, we demonstrate for an attention capture paradigm that it is, never the less, possible to extract information about the relative timing of regional brain activity during cognitive processes on the scale of 100 ms by comparing alternative signal models representing early versus late activation. We demonstrate that model selection is not driven by confounding regional differences in hemodynamic delay. We show, including replication, that the activity in the dorsal anterior insula is an early signal predictive of behavioral performance, while amygdala and ventral anterior insula signals are not. This specific finding provides new insights into how the brain assigns salience to stimuli and emphasizes the role of the dorsal anterior insula in this context. The general analytic approach, named “Cognitive Timing through Model Comparison” (CTMC), offers an exciting and novel method to identify functional brain subunits and their causal interactions.

## Graphical abstract

The temporal specificity of functional magnetic resonance imaging (fMRI) is limited by a sluggish and locally variable hemodynamic response trailing the neural activity by seconds. Here, we demonstrate that it is, never the less, possible to extract information about the timing of cognitive processes on the scale of 100 ms by comparing alternative signal models. Using this technique for an attentional capture paradigm, we show, including replication, that the activity in the dorsal anterior insula is an early signal predictive of behavioral performance, while amygdala and ventral anterior insula signals are not.

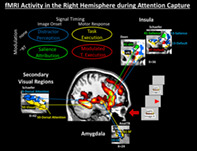

## 1 INTRODUCTION

Functional magnetic resonance imaging (fMRI) is a widely used method to identify the source of neural activity inside the brain with high spatial resolution (~1 mm) by observing subsequent hemodynamic changes such as increased blood flow. However, the temporal resolution of the technique is often considered to be poor because hemodynamic changes follow neural activity in a sluggish manner with a spatially variable delay of 4–6 s. Relevant neural interactions detected by electrophysiological methods such as electroencephalography (EEG) occur at two orders of magnitude smaller time scales (~50 ms). Therefore, we cannot easily investigate the relative timing, that is, causality, of neural activity between regions with fMRI. Overcoming this limitation would be a major advance for brain science. In this study, we are exploiting the trial‐by‐trial differences in timing of cognitive events from the presentation of a stimulus to the subsequent motor response to achieve this. We demonstrate that comparing alternative signal models (Soch & Allefeld, 2018) can reliably separate signals associated with the initial stimulus onset from later motor response‐related signals because reaction times (RTs) vary with a standard deviation on the order of 70 ms. This is not possible using the conventional general linear modelling (GLM) approach and is not driven by spatially variable hemodynamic response delays (Appendix S1). Our analysis yields a highly informative map of brain activity that differentiates cognitive (functional) subprocesses corresponding to different fMRI signal models.

**FIGURE 1:**
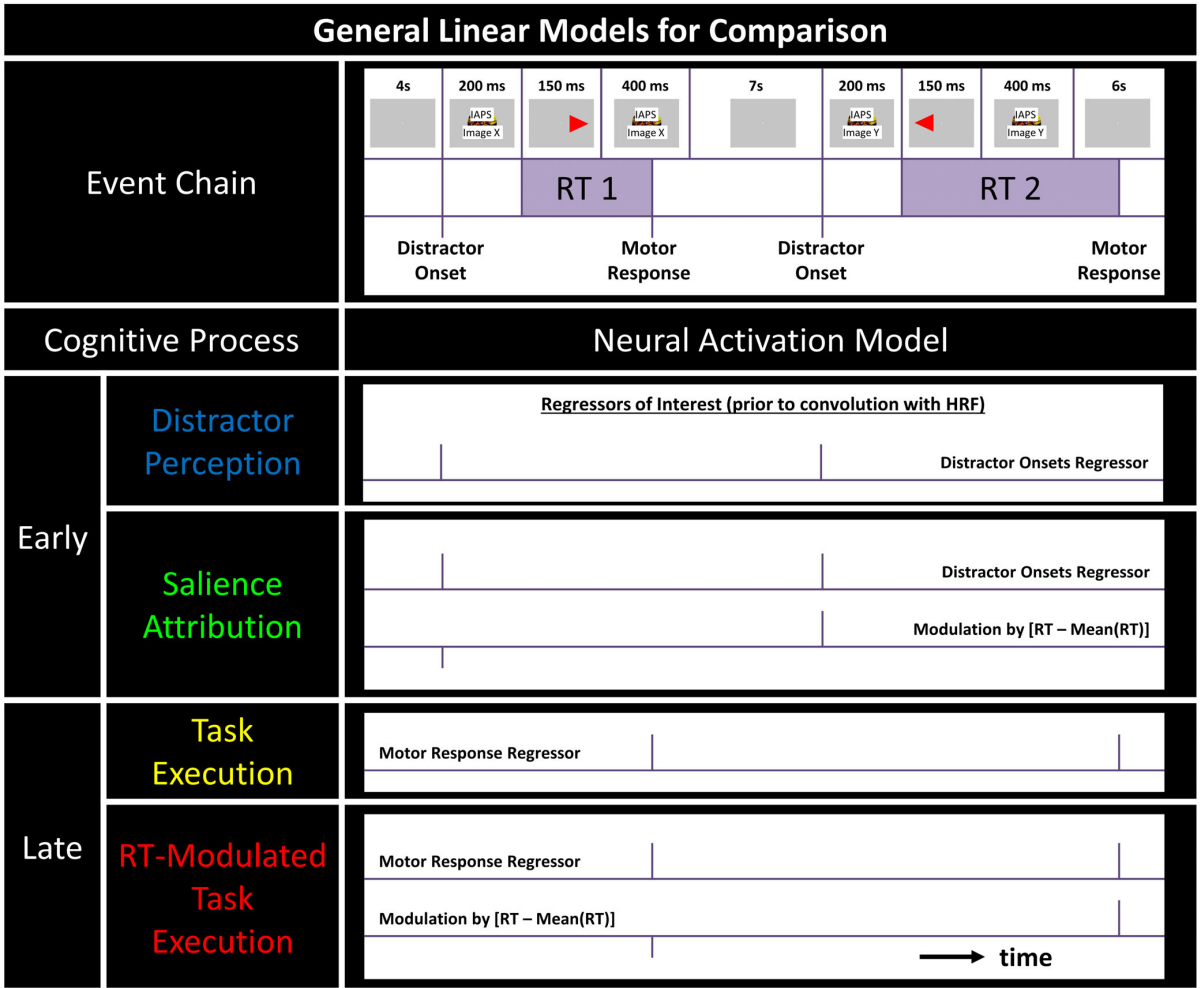
Illustration of the ACES task, the related cognitive processes and the associated fMRI models. The temporal sequence of cognitive events is shown for two trials in the top row (time is not to scale). We are describing four distinct processes. Initially in each trial, the stimuli (distractor and task cues) will be perceived by the visual system (*distractor perception*). Very quickly and automatically, the brain has to evaluate the relevance of the distractor (*salience attribution*). Later in the process, the brain has to prepare and execute the correct response (*task execution* and *RT‐modulated task execution*). Importantly, RTs vary in each trial. While the two early processes can be expected to be time‐locked to the onset of the distractor images, the later events will be more closely associated with the response execution. Only the *salience attribution* and *RT‐modulated task execution* model include a modulation by RT (deviation from the mean) and, thus, potentially contain information about the salience (distraction power) of the distractor image. Note that the neural events will be convolved with a broad HRF to obtain the actual fMRI data model (not shown). Identifying the best‐fitting model in a particular voxel of the brain comprises evidence that this brain region may be involved in the associated cognitive process. ACES, attention capture by emotional stimuli; HRF, hemodynamic response function; RT, reaction time

**FIGURE 2:**
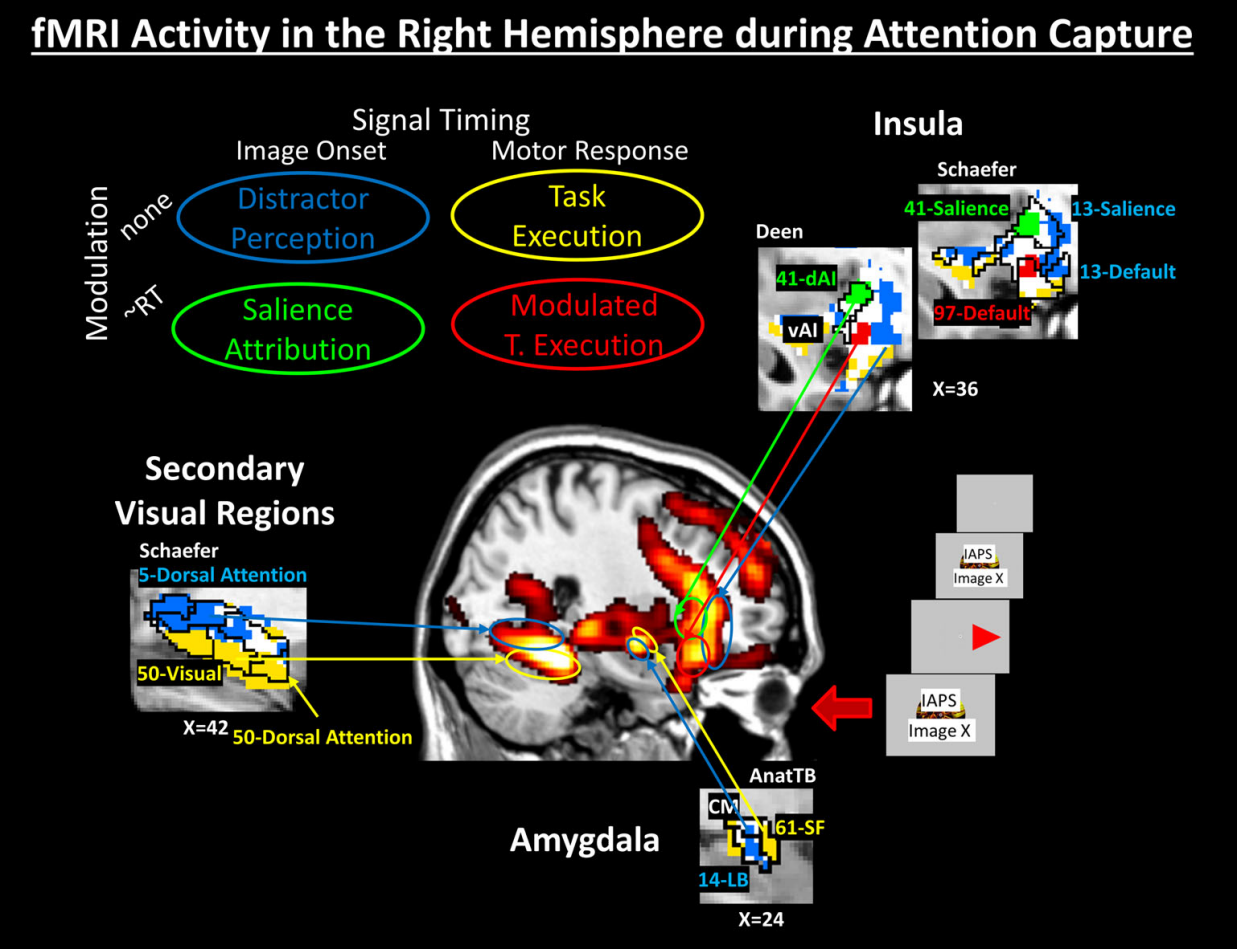
An illustration of core brain regions that are known to be associated with ACES: Amygdala (CM, SF, and LB), dAI and vAI, secondary visual areas (see Appendix S1). The central heat map image is a maximum intensity projection through the right hemisphere of the fMRI signal difference negative > neutral distractors (*p* < .001 uncor. group data for *N* = 40) overlaid on a sagittal slice of a T1‐weighted structural brain scan. For each of these regions, winning models are presented in the cutouts for a single slice at the given sagittal slice position in MNI space. The different colors represent the winning model over all participants within each region as indicated in the upper left. White regions were not replicable. Events within the models (Figure 1) are time‐locked either to the image onset or to the motor response and they may or may not show an amplitude modulation with RT as a measure of behavioral performance. Regional boundaries are drawn based on different brain atlases (Schaefer, Deen or Anatomy Toolbox [AnatTB]; see Appendix S1). Winning model clusters are labeled with their cluster number and the respective atlas label (functional network). ACES, attention capture by emotional stimuli; CM, centromedial; dAI, dorsal anterior insula; LB, laterobasal; RT, reaction time; SF, superficial; vAI, ventral anterior insula

We are demonstrating this methodological innovation in the context of an attention capture by emotional stimuli (ACES) task (Marxen et al., 2021). Humans and animals must constantly monitor and evaluate their surroundings to survive and potentially adapt their behavior to a given situation. When we walk through the jungle, it is important to differentiate quickly between a tree branch and a snake. Functional impairments in everyday life or even mental disorders, such as depression, may arise when this process is not well‐balanced (Pilhatsch et al., 2014). In our task, participants are presented with a left‐ or right‐pointing triangle, preceded and followed by a very brief display of a distractor image (Figure 1 and [Marxen et al., 2021]). Subjects have to indicate as quickly as possible the pointing direction of the triangle by a button press and will respond slower, in average, when the distractor image is perceived as negative in valence (threatening, disgusting, etc.) as compared to neutral (Gupta et al., 2016; Padmala et al., 2017). This response slowing is thought to reflect a withdrawal of limited cognitive resources from executing the task towards evaluating the potential threat (Marxen et al., 2021; Schmidt et al., 2015). fMRI studies show a well‐known, extended network of brain regions that react stronger to negative emotional images as compared to neutral images (Figure 2 and [Dolcos et al., 2011; Marxen et al., 2021]). However, we do not really understand the specific role of each particular region within this network during the sequential process of perceiving the stimulus, attributing salience (significance) to the stimulus and executing a response. For example, it is an open question whether the amygdala or the insula, which are both part of this network, may be responsible for the attribution of salience to a particular distractor (Carretie, 2014; Marxen et al., 2021; Uddin, 2015). We hypothesized that salience attribution occurs primarily in the dorsal part of the anterior insula (dAI) and not in the ventral anterior insula (vAI) or amygdala based on our previous study (Marxen et al., 2021) and existing literature on insula subdivisions (Deen et al., 2011; V. Menon et al., 2020; Uddin, 2015). A salience attribution region should satisfy three conditions: 1. It should be able to separate negative from neutral distractors, that is, be part of the above network; 2. It should carry information about the RT within a trial, which is the behavioral measure of distractor salience; and 3. It should respond to the stimuli early in the processing chain as it needs to guide the subsequent distribution of cognitive resources. We will demonstrate that all three aspects, especially the last one, can be addressed using fMRI, despite its sluggish hemodynamic response, using model comparison (Appendix S1). All three properties are represented in the *Salience Attribution* model described in Figure 1. We will show that identifying this model or process over alternative models/processes is possible and replicable. We will also demonstrate that our findings are supported by and supplement previous brain parcellation studies that do not utilize timing information. We are referring to our methodological approach as “Cognitive Timing through Model Comparison (CTMC).”

## 2 MATERIALS AND METHODS

### 2.1 Experimental design and regions‐of‐interest

The presented results are based on data from a currently unpublished fMRI neurofeedback study (Study B) modelled after an already published fMRI neurofeedback study (Study A) (Marxen et al., 2016). An ACES task was initially administered as part of Study A, analyzed in a conventional way, and published (Marxen et al., 2021). This previous data serves here only as a reference for replication and to provide a region‐of‐interest (ROI) for the current analysis of data from an independent subject sample. This ROI was defined as the intersection of the previously published negative > neutral distractor fMRI contrast (*p* < .001 uncor.) with a whole brain mask. This whole brain mask was computed as the intersection of three whole brain masks from the SPM analyses conducted in Study A (one mask) and B (two masks from two sessions). For the whole brain analyses of Study B, the intersection of the latter two masks was used.

In Study B, the ACES task was administered on the first and last of five experimental days. On the first day, it was acquired after a structural T1‐weighted MRI and a resting state fMRI sequence. After the ACES paradigm, an unrelated emotion regulation task was also acquired. On experimental days 2–4, most participants undertook an emotion regulation training with or without real‐time fMRI neurofeedback. On the last (fifth) experimental day, the ACES task followed two runs associated with the emotion regulation training. Again, the ACES task was followed by the emotion regulation task from day 1. For the purpose of this study, the data acquired on experimental day 5 serves as replication data for the results obtained on experimental day 1. Effects of the regulation training are not assessed in this context. The median time between the first and last experimental day was 30 days (min: 9, max: 111) for *N* = 33 analyzed data sets (see Section 2.2).

### 2.2 Participants

Study A: *N* = 44 participants were analyzed for the behavioral data, *N* = 40 for the fMRI contrast. See (Marxen et al., 2021) for more details.

Study B: Forty‐three additional healthy, right‐handed (Edinburgh Handedness Inventory score > = 50) adults in the age range of 18–40 years were recruited using the same criteria as in Study A. All participants were screened for exclusion criteria including a history of mental disorder, physical conditions that prevent lying comfortably inside an MRI scanner, a body mass larger than 120 kg, further MRI contra‐indications, vision impairments outside of −5 to +3 diopters, insufficient knowledge of the German language, and pregnancy. Due to technical issues in recording the behavioral responses in two participants and the abortion of the task by one participant, data of three participants were excluded, leaving *N* = 40 participants (18–38 of age, mean = 24.28, SD = 3.95, 12 males) for the behavioral and fMRI analyses of session 1 (B1). In session 1, no participant had more than 10 missed or incorrect responses in 120 trials or more than 7.5% of fMRI volumes with a frame‐wise displacement (FD) >0.5 mm (Power et al., 2012). Of these *N* = 40 participants in session 1, *N* = 39 completed session 2. Of these, four had to be excluded because they had more than 10 missed or incorrect responses in session 2. Two more participants had to be excluded for the fMRI analysis because they had more than 7.5% of fMRI volumes with a FD >0.5 mm in session 2. Hence, *N* = 35 participants from session 2 qualified for the behavioral analysis and *N* = 33 participants for the fMRI analysis (B2). For participation in all five sessions, participants received a compensation of €90.00 on the last experimental day. All participants signed informed consent forms after receiving a detailed description of the study. The study was approved by the Ethics Review Board of the Technische Universität Dresden (EK 56022012) and carried out at the Neuroimaging Center of the Technische Universität Dresden.

### 2.3 The attentional capture by emotional stimuli task

In the ACES task, participants performed a simple choice RT task in between the display of images with either positive, neutral, or negative valence. The task has previously been described in Marxen et al. (2021) and operationalizes valence effects of emotional distractor images on a cued RT task (or “attentional capture”) as response slowing. As illustrated in Figure 1, each trial begins with the display of an (emotional) image in the center of the screen for 200 ms. This image is followed by a 150 ms presentation of a red triangle either pointing right and positioned to the right of the center of the screen or pointing left and positioned to the left of the center of the screen. The trial concludes with a 400 ms display of the same image that preceded the red triangle. Participants were instructed to press as fast as possible a left button with their right index finger for the left pointing triangle and a right button with their right middle finger for the right pointing triangle. The full task consisted of 120 trials with 40 trials for each valence category and separated by an inter‐stimulus‐interval (ISI) of 4, 5, 6, or 7 s (*M* = 5.5 s). Negative, neutral, and positive distractor images were mostly taken from the International Affective Picture System (IAPS) (Lang et al., 2008). Across participants, five different presentation orders of distractor images and ISIs were used. Valence categories for female/male participants were categorized according to female/male IAPS ratings. A table with the used images and valence ratings can be found in the supplemental material to Marxen et al. (2021). The average rating (mean ± SD) for negative/neutral/positive images was 1.9 ± 0.7/5.6 ± 0.7/7.5 ± 0.5 and 2.6 ± 0.7/5.4 ± 0.6/7.0 ± 0.4 for females and males, respectively. All images were modified such that the primary stimulus was cut out circularly and occupied a visual angle of 9° for the participant to avoid additional response slowing related to visual search phenomena. Images and triangles were presented on a grey background with a black central fixation cross. The experiment was programmed in Presentation® software (Version 16.3, Neurobehavioral Systems Inc.).

### 2.4 Functional MRI data acquisition (Study B)

All MRI data were obtained using a 3‐Tesla Siemens Tim Trio scanner with the Siemens 32‐channel head coil (Siemens). T1‐weighted images were acquired with a 3D magnetization‐prepared rapid gradient echo (MPRAGE) sequence (repetition time [TR] = 1.9 s, echo time [TE] = 2.26 ms, field of view [FOV] = 256 × 224 × 176 mm^3^, voxel size = 1 × 1 × 1 mm^3^, inversion time = 0.9 s, flip angle [FA] = 9°, phase partial Fourier 7/8, bandwidth [BW] = 200 Hz/Px). Functional data (1059 volumes) of the ACES task were acquired with the CMRR multi‐band EPI sequence (Moeller et al., 2010; Setsompop et al., 2012) with multi‐band factor 6, TR = 756 ms, TE = 25.0 ms, FOV = 195 × 195 × 144 mm^3^, voxel size = 2.5 × 2.5 × 2 mm^3^, no slice gap, 72 interleaved slices tilted mildly from axial to coronal, flip angle = 55°, 7/8 phase partial Fourier, weak raw data low‐pass filter, band width = 2466 Hz/Px.

### 2.5 Analysis of behavioral data—reaction times and error rates

Behavioral data were analyzed using MATLAB R2018b (The Mathworks, Inc.). To replicate findings related to the behavioral emotional distractor effect by Marxen et al. (2021), the same statistical analyses were performed on the present data set. For each participant, the mean RT of correct responses (i.e., correct directional response within 1600 ms after target onset) and error rate (i.e., percentage of trials with incorrect responses or no response within 1600 ms after target onset) for the three valence categories was calculated. Since the first and second trial have been found to be outliers by Marxen et al. (2021), they were excluded from analysis. To test for an effect of distractor valence on RTs and error rates, two separate repeated‐measures ANOVAs with the within‐subject factor valence (negative [neg], positive [pos], and neutral [neu]) were performed. Significant RT effects were followed by one‐sided paired *t* tests for mean(RTneg) > mean(RTneu), mean(RTneg) > mean(RTpos), and mean(RTpos) > mean(RTneu) to address the direction of the distractor effect. Uncorrected *p*‐values, group mean RT values with standard errors, Cohen's *d* = (mean(RTneg) − mean(RTneu))/sqrt(mean(var(RTneg), var(RTneu))), and Cohen's *dz* = mean(ΔRTneg‐neu)/std(ΔRTneg‐neu) as a measure of the distractor effect size are reported. Additionally, 95% confidence intervals (CIs) were calculated based on 1000 bootstrap samples.

### 2.6 Analysis of functional MRI data: preprocessing

The fMRI data were preprocessed using SPM8 (Wellcome Department of Cognitive Neurology). First, functional EPI images were realigned, and distortion (field map) corrected using a voxel displacement map calculated from presubtracted phase and magnitude data. Next, the mean EPI image was coregistered to each participant's T1‐weighted MR image using the normalized mutual information algorithm. T1‐weighted images were further segmented and then normalized to the MNI space. Using the transform parameters of the normalization of the T1‐weighted image, functional images were normalized to MNI space and resampled into 2 mm isotropic voxels. Finally, normalized images were spatially smoothed with a Gaussian kernel with an FWHM of 6 × 6 × 6 mm.

### 2.7 Analysis of functional MRI data: subject‐level general linear models

For subsequent model comparison, four different subject‐level GLMs were computed (Figure 1) using SPM12. Two of these with events time‐locked to the distractor image onset (Early processes) and the other two with events time‐locked to motor response (Late processes). To test whether local fMRI signals predict RTs, a mean‐free parametric RT regressor was included optionally in each model class. Note that RT values below the mean result in negative spikes in the RT regressor and imply for a positive regressor coefficient that the event‐related activation is reduced compared to the mean activation. This resulted in 2 (distractor onset vs. motor response regressor) × 2 (with vs. without parametric RT regressor) models (see table in Figure 2). Following Figure 1, each model was associated with one of four cognitive processes: *Distractor Perception*, *Salience Attribution*, *Task Execution*, and *RT‐modulated Task Execution*. Note, that the *Salience Attribution* model is identical to the RT model in (Marxen et al., 2021) (see also Replication of Study A). Furthermore, single event regressors for missed or incorrect trials and six motion regressors were included in all models. Regressors, except for the motion regressors, were convolved with SPM's canonical hemodynamic response function (HRF). A high‐pass filter at *T* = 128 s and a first‐order auto‐regressive AR(1) model was chosen for temporal filtering and non‐sphericity correction, respectively. In a supplemental analysis, four additional models were computed by adding the first‐order temporal derivatives to the event regressors (see Model Comparison) to investigate the effect of a variable latency of the HRF (see below), which is approximated by non‐zero derivative regression coefficients (Calhoun et al., 2004).

### 2.8 Analysis of functional MRI data: model comparison approach

**FIGURE 3:**
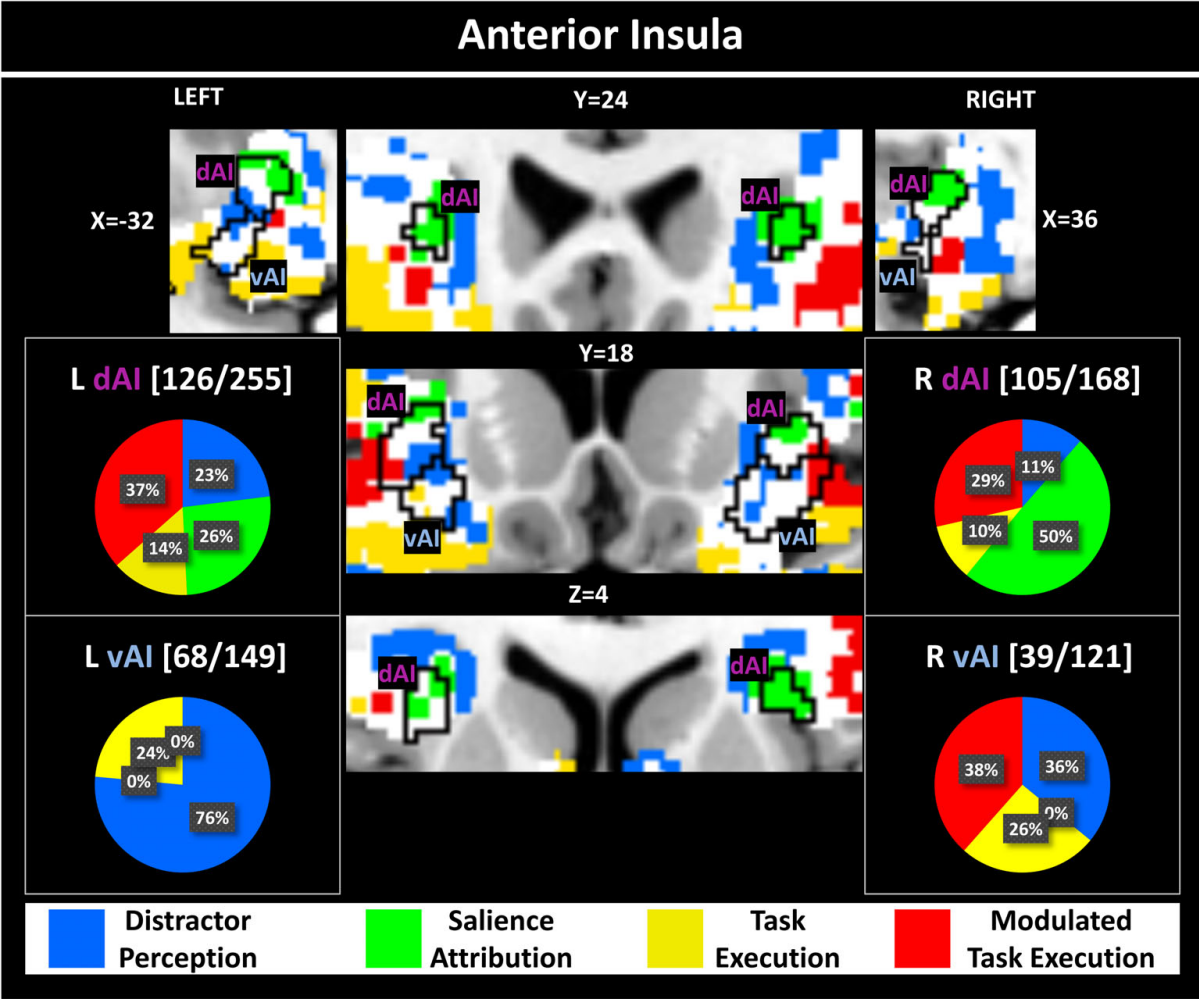
Winning model maps for the anterior insula region. MNI coordinates are given in mm. The color scheme is the same as in Figure 2. Boundaries from the Deen atlas (Deen et al., 2011) (intersections with the negative > neutral contrast) are shown for the dAI and vAI. The pie charts indicate the percentage of all replicable voxels within these subdivisions that belong to a particular model. Above each chart, the numbers of replicable voxels with the subdivision and the size of the subdivision are given, which is equivalent to the following reproducibilities: Left dAI—49%, left vAI—46%, right dAI—63%, right vAI—32%. Note that sizes of the subdivisions reflect the intersection of the atlas region with the negative > neutral contrast, not the size of the complete atlas region (given in Appendix S1). dAI, dorsal anterior insula; vAI, ventral anterior insula

**FIGURE 4:**
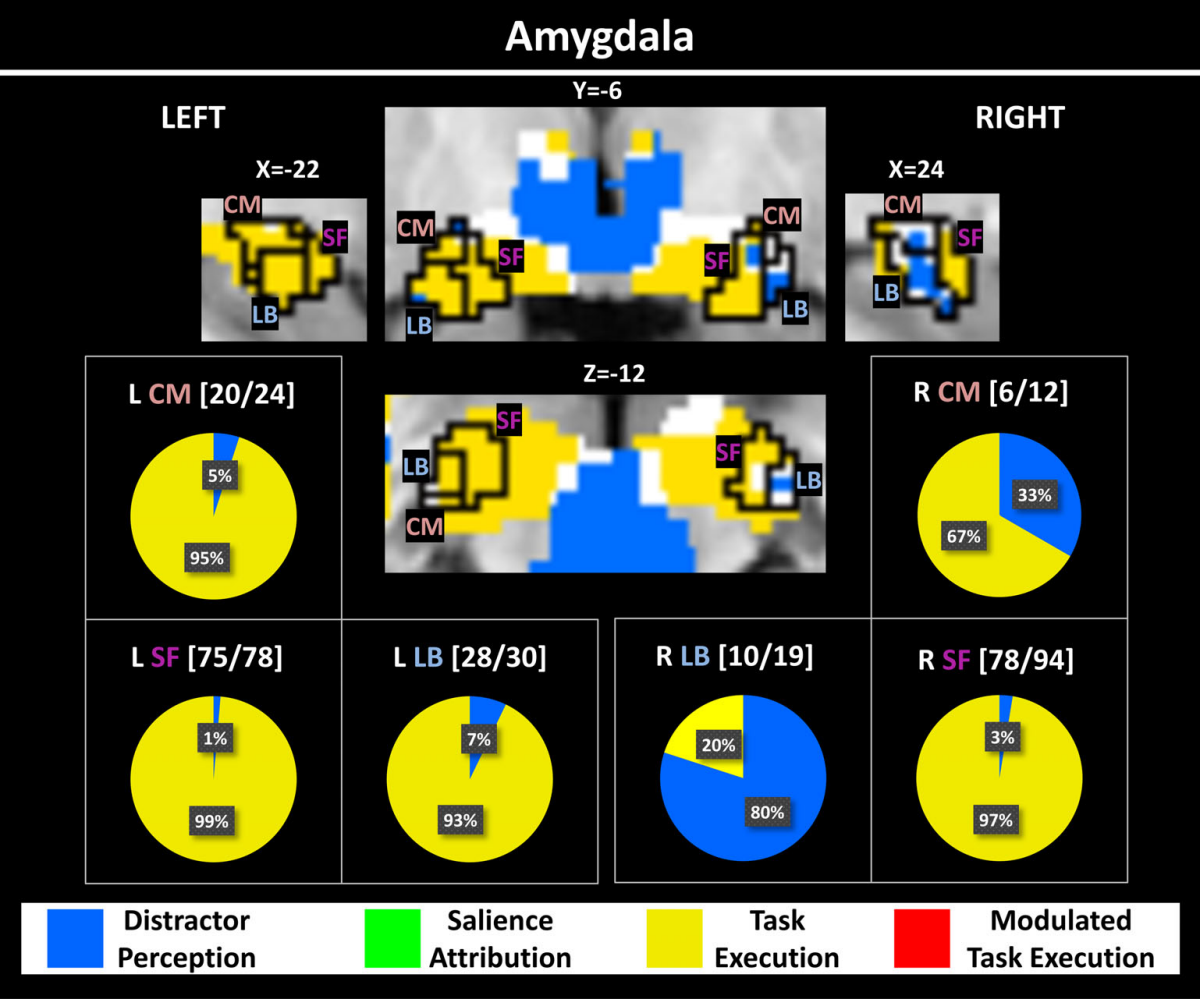
Winning model maps for the amygdala region. MNI coordinates are given in mm. The color scheme is the same as in Figure 2. Boundaries from the SPM anatomy toolbox version 1.8 (intersections with the negative > neutral contrast) are shown for the CM, the SF, and the LB subdivisions of the amygdala. The pie charts indicate the percentage of all replicable voxels within a subdivision that belong to a particular model. In the amygdala, only two of the four models occur. Above each chart, the number of replicable voxels within the subdivision and the total size of the subdivision are provided. This information is equivalent to the following replicability: Left CM—83%, left SF—96%, left LB—93%, right CM—50%, right SF—83%, right LB—53%. CM, centro‐medical; LB, laterobasal; SF, superficial

To assign brain regions to a particular model, that is, one of four cognitive processes, we utilized the cross‐validated Bayesian model selection (cvBMS) approach implemented in the MACS (Model Assessment, Comparison, and Selection) toolbox for SPM (Soch & Allefeld, 2018), which includes the following steps. First, a model space is specified of the candidate models to be compared. Cross‐validated log model evidence (cvLME) maps were estimated on a subject‐level for each model. For each participant, these maps quantify the models' performance in each voxel of the brain. Then, on the group level, a random‐effects Bayesian model selection (Stephan et al., 2009) was applied to the subject‐level cvLME maps. This results in group‐level, voxel‐wise brain maps of likeliest frequencies (LFs) and exceedance probabilities (EPs) for each model visualizing how well a model performs relative to any other model in the model space. LFs range between 0 and 1 and can be interpreted as the proportion of participants in which the corresponding model is the optimal one. EPs also range between 0 and 1, but quantify the probability that a model is superior to the other models. Both parameters lead to the same model ranking (Soch et al., 2016; Stephan et al., 2009), which was used to generate winning‐model maps using Matlab R2019b (The Mathworks, Inc.) indexing the winning model in the above order. For visualization, these indexes were translated into color‐coding following the color scheme in Figures 2, 3, and 4. To restrict reported winning models to replicable regions, the same analysis was conducted twice (Analysis B1 and B2) and not replicable voxels were identified and displayed in white. In this article, this replicably‐winning‐model map is only shown within the ROI mask of the negative > neutral distractors contrast.

To investigate the possibility that the later models are only selected because of the fact that responses are delayed by approximately 600 ms (see Appendix S1—Supplementary Description of Modeling Approach) from the distractor onset, we repeated the above analysis with eight models that included temporal derivative regressors, which mimic altered HRF latencies (Calhoun et al., 2004). In case that the 4‐model approach assigns a region only to the late processes because of longer latencies of the HRF but is truly associated with the distractor onset, the early models plus derivatives should outperform the late models without derivatives.

### 2.9 Analysis of functional MRI data: calculation of replicability

Based on the two replicably‐winning‐model maps from analysis B1 and B2, replicability in percentage was computed as the ratio of replicable voxels between the two analyses to the total number of voxels in the ROI/whole brain times 100. As this number is expected to rise for larger group sizes *N* for values above chance due to an increase in power, we also computed the average within‐subject replicability including the 95% CI using 1000 bootstrapping samples from the 33 subjects of analysis B1 that are also available in study B2. We consider replicability as better than chance if the chance level of 25% is not included in the CI.

### 2.10 Analysis of functional MRI data: brain atlases used for comparison

As CTMC is a new approach to functionally parcellate the brain, we utilized six state‐of‐the‐art brain atlases for comparative purposes: 1. the fMRI functional connectivity network parcellations by Schaefer with 400 parcels (Schaefer et al., 2018); 2. an amygdala atlas based on cytoarchitectonics and probability maps (probability > 0.5) provided with the SPM Anatomy Toolbox 1.8 (Eickhoff et al., 2005); 3. the fMRI functional connectivity‐based insula atlas by Deen et al. (2011); 4. The automated anatomical labeling AAL3v1 atlas (Rolls et al., 2020; Tzourio‐Mazoyer et al., 2002), which is based on anatomical MRI scans; 5. a modified version of the multi‐modal brain atlas (360 regions) by Glasser et al. (2016), which is based on both functional and anatomical MRI data. The Horn version of this atlas was modified for volumetric analysis in the MNI space by assigning all voxels within the Schaefer atlas above to Glasser labels using a nearest‐neighbor approach; 6. the Neuromorphometrics atlas contained in SPM12, which is built from a database of neuroanatomically labeled MRI scans.

This article refers to the first three atlases only. The additional three atlases were included for expert readers in Appendix S1 and utilized in Figure S1. For quantitative purposes, all atlases were resampled to the same resolution as the winning‐model maps (2 mm). For visual display purposes, the atlases were resampled to 0.5 mm resolution using nearest‐neighbor interpolation, and regional boundary maps were generated by relabeling voxels with “0” if all 26 neighboring voxels had the same label and “1” otherwise. For the analysis restricted to the negative > neutral distractor contrast ROI, atlases and subsequently boundaries were limited to the ROI. Note, that this means that all boundaries in Figures 2, 3, 4 are restricted to stay within the negative > neutral distractor ROI.

### 2.11 Analysis of functional MRI data: reporting of winning model results

To report the results contained in the replicably‐winning‐model map in a quantitative way and place them into the context of existing brain parcellations, the maps were clustered for each model using SPM12 (the map with the cluster indices is provided), and two data tables were generated: one for the negative > neutral distractor ROI and one for the whole‐brain mask. These tables contain information about each cluster and how much it overlaps with a particular atlas region. Specifically, these tables contain:

1. the index of the cluster,
2. the model number ordered as in Figure 1,
3. the cluster size in voxels ([2 mm]^3^),
4. the center of mass (CoM) of the cluster in MNI space,
5. the name of one of the above atlases,
6. the name of the atlas region,
7. the number of voxels that belong to the intersection of the cluster with the atlas region,
8. the percentage of (7) with respect to (3),
9. the number of voxels that belong to the intersection of the atlas region with the negative > neutral contrast ROI
10. the percentage of (7) with respect to (9)
11. the original number of voxels inside the atlas region
12. the percentage of (7) with respect to (11)

In addition, we are providing a manual to view the maps and to identify the index of the corresponding cluster and the name of the atlas region (see Supplementary Data S1 at https://osf.io/kqz6a/).

### 2.12 Analysis of functional MRI data: Replication of Study A

The behavioral analysis of Study A was replicated in Study B following the above section Analysis of Behavioral Data: RTs and Error Rates. Results for RTs are presented in Table 1 of the main manuscript. Results for error rates are presented in Table S1. In addition, we replicated the Valence and RT—Models of Study A as conducted in (Marxen et al., 2021) for the *N* = 40 subjects of Study B—Session 1. On the subject level, the Valence Model included three regressors, each for one of the three valence categories (negative, neutral, and positive). On the group level, a full factorial model with the three‐level factor valence was used to test for differences in fMRI activity for negative compared to neutral distractors of the Valence Model. The RT Model is identical to the *Salience Attribution* model of the current study. A random effects analysis using a one‐sample *t* test was applied to regression coefficients of the parametric RT regressor of the model. A cluster threshold of *p* < .001 (no minimum cluster size) and family‐wise error correction (FWE) for a small volume (SVC) with pFWE <.05 using the bilateral AMY and AI masks (Marxen et al., 2021) was used.

**TABLE 1:** Reaction time results for negative and neutral distractors

		$\bar{\mathrm{RTs}}$ (SE)		Neg vs. Neu			
	N	Neg	Neu	Neg‐Neu	*t*	*d* (95% CI)	*d*_*z*_ (95% CI)
Study A	44	430 ± 14	410 ± 12	19.44 ± 1.95	3.99**	0.23 (0.13–0.45)	0.60 (0.35–0.90)
Study B1	40	398 ± 9	385 ± 8	12.33 ± 1.64	4.41**	0.23 (0.09–0.36)	0.70 (0.33–1.03)
Study B2	35	384 ± 9	376 ± 8	7.75 ± 1.71	1.79*	0.15 (−0.05–0.33)	0.30 (−0.12–0.67)

*Note*: Group mean RTs are presented with SEs, results of one‐sided paired *t* tests and the effect sizes Cohen's *d* and *d*_*z*_ with 95% CIs.

Abbreviations: CI, confidence interval; Neg, negative; Neu, neutral; RT, reaction time; SE, standard error.

* *p* < .05.

** *p* < .0005.

## 3 RESULTS

### 3.1 Behavioral results

Detailed behavioral results are presented in Table 1 for RTs and Table S1 for error rates. Relevant for our model comparison approach, the group mean standard deviation of the RTs was 72 ± 5 ms (SE—standard error of the mean) in Study B1 and 78 ± 6 ms in Study B2.

### 3.2 Functional MRI results

We compared four models (Figure 1) using Bayesian model comparison (Soch & Allefeld, 2018). On the group level, a map indicates the model with the likeliest frequency in each brain voxel (Figures 2, 3, 4). This “winning‐model‐map” is generated separately for Study B1 (*N* = 40) and B2 (*N* = 33) to examine replicability. In Figure 2, replicable regions are shown in color, not replicable ones in white. The map is restricted to the negative > neutral contrast brain mask derived from Study A. The four compared models can be arranged in a 2 × 2 matrix as shown in the upper left corner of Figure 2 and are associated with the cognitive processes *Distractor Perception*, *Salience Attribution*, *Task Execution*, and *Modulated Task Execution*. Please refer to the figure caption and Section 4 for more details.

### 3.3 Replicability of winning model

Within the group‐level winning model map, of 17,758 voxels within the mask, 65% are replicable. On the whole‐brain level, 64% of 217,841 voxels are replicable. Inside the mask, we identified 106 (51 with a minimum size of 10 voxels or 80 mm^3^) replicable clusters, 39 (20) for *Distractor Perception*, 9 (4) for *Salience Attribution*, 44 (21) for *Task Execution*, and 14 (6) for *Modulated Task Execution*. The average within‐subject replicability (*N* = 33) was 36 (95% CI: 34–38) % in the mask and 36 (34–39) % in the whole brain (chance level 25%). Note that the group‐level replicability values above are expected to rise with *N* due to higher statistical power.

### 3.4 Winning models within anterior insula

Figure 3 shows the results of the model comparison for the anterior insula. Clusters of *Salience Attribution* can be identified only in the left and right dAI, while *Modulated Task Execution* signals also occur in the right vAI extending towards more frontal and lateral regions.

### 3.5 Winning models within the amygdala

Figure 4 shows the results of the model comparison for the amygdala. The best fits were obtained either with the *Distractor Perception* model or the *Task Execution* model. The two RT‐modulated models did not occur.

### 3.6 Correspondence with anatomical and functional brain parcellations

Winning model maps need to be viewed in the context of existing brain parcellations to evaluate whether they are congruent with known functional boundaries. To this end, images of replicable clusters and lists including the winning model, cluster size, center‐of‐mass in MNI space, and overlap with regions from six different brain atlases are given in Appendix S1 for the negative > neutral distractor contrast as well as for the whole brain. As this data is extremely rich, for the purpose of this article, we are focusing our discussion on the brain regions shown in Figures 2, 3, 4. The secondary visual region was included in Figure 2 to demonstrate corresponds between boundaries in the winning model map with known boundaries in functional networks (see Section 4). A more detailed account of the data is provided in the Supplementary Results (Figure S1). The following atlases were utilized in these figures: 1. the functional connectivity network parcellations by Schaefer et al. with 400 parcels (Schaefer et al., 2018); 2. an amygdala atlas based on the SPM Anatomy Toolbox 1.8; 3. a functional insula atlas based on connectivity‐analyses by Deen et al. (2011).

### 3.7 Analysis of functional MRI data: Replication of Study A

Figure S6 and Table S2 show the results of the replication of Study A in Study B1.

## 4 DISCUSSION

Our discussion will focus on three major findings: First, that model comparison for the analysis of task‐based fMRI data can reveal timing information in cognitive processes on the time scale of 100 ms. Second, that the dAI is not only a key region during the processing of distractor stimuli, but may specifically mediate the behavioral correlates of attentional capture. Third, that the CTMC‐based functional parcellation of the brain is in line with and an extension of existing functional and anatomical brain parcellations.

Because of the nature of the HRF, the temporal resolution of fMRI is considered poor, which is a critical limitation of this spatially precise neuroscientific method. Note that broadness and delay of the HRF are not a principle hindrance to measure timing differences between regions with high precision as temporal shifts of the rising edge of the response can be detected rather precisely. In fact, Menon emphasized the importance and the opportunity of using fMRI for the study of temporal processing in the brain and employed the term “mental chronometry” (Menon, 2012; Menon et al., 1998). Consistent with this notion, Katwal et al. have demonstrated that relative timing differences of 28 ms could be detected in visual cortex (Katwal et al., 2013). However, the problem of the hemodynamic response with regard to cognitive neuroscience is less its precision within a particular voxel and participant but rather its variability in latency across the brain of at least ±1 s (Chang et al., 2008; Miezin et al., 2000), an order of magnitude above relevant cognitive time scales! Such local variation in the neuro‐vascular coupling process fundamentally limit our ability to draw conclusions about the relative timing on the neural level and causal effects (that presume temporal precedence) between different brain regions. This critical perspective of causal (effective) functional connectivity methods is well known in the field (Lohmann et al., 2012; Smith et al., 2012). Deconvolution techniques (Aquino et al., 2014; Bush et al., 2015; Stephan & Roebroeck, 2012) are a possible solution to the problem, but require independent information about local neural activity (usually unavailable) to be unbiased, are susceptible to noise and the parametrization of the deconvolution kernel (Aquino et al., 2014; Bush et al., 2015).

Our CTMC approach is not susceptible to such details of the hemodynamic response because we are inferring neural timing from trial‐by‐trial timing variations between stimulus onsets and motor responses and use fMRI model comparison to identify the associated cognitive process. The pivotal point here is the discovery that the standard deviation of ~70 ms in the behavioral response times is sufficient to identify through Bayesian model comparison whether a local BOLD signal is associated with an early (distractor processing) or late (task execution) cognitive process. Note that including variations of the hemodynamic delay into the models through temporal derivative terms has only a marginal impact on the results (see Figure S4 and Supplementary Text and Description of Modeling Approach in Appendix S1). Other approaches to model spatially variable HRFs, such as selecting from a library of potential HRF shapes (Prince et al., 2022), could be investigated in this context but would require many more model comparisons and would require additional choices such as how to select the correct neural onset for HRF selection.

Two lines of argumentation show that associating a model with a particular cognitive process is possible and informative: First, the winning model map is replicable in ~64% of all voxels (Figure S3) and in average in 36% of all voxels on the subject‐level with a CI of 34–39%. Given a chance level of 25%, it is, therefore, not a noise signal. Second, neighboring, replicable clusters of different winning models are in many cases directly touching, for example at the boundary between the dorsal attention network and the visual network (Figure 2). These sharp spatial boundaries indicate consistency between subjects (after mapping into the common MNI space) and good agreement with existing knowledge of localized brain functions (see also Supplementary Results in Appendix S1). Note that the Schaefer atlas labels the most inferior and anterior region of the yellow *Task Execution* cluster in Figure 2 as part of the Dorsal Attention network. Our analysis indicates that it may be more appropriate to consider this parcel part of the visual network.

Here, we employed CTMC to study the functional role of brain regions during ACES. We addressed in particular the question which brain region might be central to the attribution of distractor salience. To this end, we specified one model that identifies brain regions that meet all three requirements of *Salience Attribution* (see Section 1), and three alternative models, which either lack the requirement of early activation (the Modulated and unmodulated *Task Execution* Models) or modulation by RT (pure *Distractor Perception*). Please, note, however, that CTMC is still limited by the experimental design and that an association of a given signal model with a particular cognitive process can be challenging (see Supplementary Text in Appendix S1).

Previously, we observed that while both amygdala and anterior insula (AI) show stronger BOLD signals for distractors with negative valence as compared to neutral ones, only the signal in the AI is correlated with (i.e., modulated by) the behavioral read‐out (i.e., RT). CTMC advances this previous finding by demonstrating that only the modulated signal in the dAI is associated with the distractor onset (Figure 3), thus, predictive of behavioral performance. Therefore, we conclude that the dAI in particular is central to the *Salience Attribution* process. This conclusion fits well with the assumption that salience attribution may require transitions in brain networks and is in line with recent findings of a regulatory role of the AI in precisely these transitions, for example in switching between the default mode and the executive control network (Sridharan et al., 2008) or the dorsal attention network (Huang et al., 2021). In addition, CTMC revealed that, although the ventral AI (vAI) in the right hemisphere also contains modulated signals correlated with RT, these occur later in the process (see *Modulated Task Execution* process). Thus, these vAI signals may contribute to longer RTs but are secondary to the early *Salience Attribution* process. The observed functional ventral‐dorsal segregation of the insula signal is in line with insula cytoarchitectonics (Augustine, 1996; Mesulam & Mufson, 1982). It is also consistent with fMRI studies linking vAI and dAI to distinct networks and functions, respectively (Deen et al., 2011; Schaefer et al., 2018). Especially the demarcations of the Schaefer atlas (Figures 2 and S1) identify the *Salience Attribution* area in the dAI precisely as part of the salience network. These findings illustrate that CMTC not only improves the temporal precision of cognitive processes measured with fMRI, but also supports and refines existing brain parcellations. Such a parcellation is especially relevant for studies that investigate the regulatory role of the AI subregions, for example in switching between brain networks.

For the amygdala, our data indicates that early *Distractor Perception* occurs primarily in the (right) latero‐basal (LB) amygdala (Figure 4), while the other subdivisions (CM and SF) are dominated by later *Task Execution* signals. This finding extends insights on functional amygdala segmentation from a previous large meta‐analysis (Bzdok et al., 2013), which showed that LB is primarily associated with significance detection in high‐level sensory input, CM with mediating attentional and motor responses as a main output center, and SF with olfactory and social information processing. Our findings also further contribute to the ongoing discussion of lateralized amygdala function (Palomero‐Gallagher & Amunts, 2021) and suggest that the right amygdala may be dominant in the early *Distractor Perception* process. Taken together, the idea that the amygdala serves as a “significance detector” (i.e., in terms of *Salience Attribution*) is not supported by our data. Thus, this earlier view on amygdala function may need to be revisited considering the possibility that, early in the cognitive process, the LB sensitively discriminates between stimulus classes but does not yet determine the actual distraction power (salience) of the stimulus. The late activation of CM and SF regions of the amygdala has not yet been reported. It may be that these regions are more strongly driven by later top‐down signals from e.g. striatal, cingulate and insula brain regions (Bzdok et al., 2013). This division of the amygdala by CTMC into at least two functionally distinct areas has important implications for effective (causal) connectivity studies (McFadyen et al., 2017; Sato et al., 2017) and resting‐state fMRI studies (Delli Pizzi et al., 2017) that treat the amygdala as a single functional region.

Beyond our focused investigation of amygdala and insula function within the ACES processes, CTMC reveals detailed temporal and spatial information about numerous other brain regions involved in the ACES processes. We provide an in‐depth presentation of these details for experts in the field in the Supplementary Text and provide a manual on viewing our results in 3D (https://osf.io/kqz6a/).

CTMC will provide a new dimension to functionally parcellate the brain according to the role a region plays in the temporal sequence of events that occur during a particular processing task. This comprises a major advance in our quest to identify causal interactions in brain networks. The technique comprises a bridge between spatially insensitive techniques with high temporal resolution, such as EEG, and conventional fMRI with its high spatial but poor temporal resolution. Common functional connectivity based brain parcellations, which usually eliminate wavelengths shorter than ~20 s, are enriched by information about process timing on a time scale of 100 ms. As cognitive science relies to large degree on tasks such as the ACES task, the stop‐signal task (Logan et al., 1984), the Stroop task (Stroop, 1935) and many others with similar variations in response times, the potential applications of CTMC are vast.

## AUTHORSHIP CONTRIBUTIONS

All authors have contributed to the drafting of the manuscript, have agreed to its content, and have agreed to be accountable for all aspects of this work. Contributions in detail: Conceptualization: Michael Marxen, Michael N. Smolka; Methodology: Michael Marxen, Johanna E. Graff, Philipp Riedel; Investigation: Michael Marxen, Johanna E. Graff; Visualization: Michael Marxen, Johanna E. Graff; Funding acquisition: Michael Marxen, Michael N. Smolka; Project administration: Michael Marxen, Michael N. Smolka; Supervision: Michael Marxen, Michael N. Smolka; Writing – original draft: Michael Marxen, Johanna E. Graff; Writing – review & editing: Michael Marxen, Johanna E. Graff, Philipp Riedel, Michael N. Smolka.

## CONFLICT OF INTEREST

All authors declare no conflict of interest.

## Supporting information


**Appendix S1:** Supporting Information.

## Data Availability

See cover page:Data availability statement: Subject‐ and group‐level data, all behavioral and imaging results, a manual to view these results, and all Matlab code are publicly available within the Open Science Foundation project “SFB940/1‐A7: Volitional Control of Brain Activity: Effects of Neurofeedback on Emotional Reactivity” (https://osf.io/6afq5/) in the component “Manuscript: Observing Cognitive Processes in Time through Functional MRI Model Comparison” (https://osf.io/rkbtf/). Original DICOM data and EPI images are currently NOT open access because the issue of “fingerprint information” within MRI‐data is a topic of ongoing, ethical debate in Germany. It is unclear whether this data can legally be considered “anonymized”. Discussions to establish institutional guidelines are ongoing.
