# Supplementary material for: Observing cognitive processes in time through functional MRI model comparison
Source: Hum Brain Mapp. 2022 Oct 26;44(4):1359–70. doi: 10.1002/hbm.26114 (PMC9921218; doi:10.1002/hbm.26114)
Supplement: Supplementary file 1 — Appendix S1: Supporting Information. [file HBM-44-1359-s001.pdf]

Supplementary Information for

Observing Cognitive Processes in Time through Functional MRI Model Comparison

Michael Marxen[1]*, Johanna E. Graff[1], Philipp Riedel[1], Michael N. Smolka[1].

[1]Department of Psychiatry, Technische Universität Dresden, Dresden, Germany

*Corresponding author: Michael Marxen, Ph.D., Section of Systems Neuroscience, Department of Psychiatry, Technische Universität Dresden, Würzburger Straße 35, 01187 Dresden

Phone: +49 351 46342212

Email: michael.marxen@tu-dresden.de

This PDF file includes:

Supplementary text
Figures S1 to S6
Tables S1 to S2
SI References

Other supplementary materials for this manuscript include the following:

Data S1 to Fig. 2 and Figs. S1-5

This data is provide publicly under the link https://osf.io/kqz6a/. This zip-archive MRICroGL_Files.zip contains pertinent 3D data files corresponding to Fig. 2 and Figs. S1-5 and the manual Manual_Viewing_ACES_CTMC_20211208.docx, which provides step-by-step instructions on how to view the above imaging results using the MRICroGL viewer.

Supplementary Information Text

Supplementary Description of Modelling Approach

As the aim of our analysis is the identification of brain regions responsible for the attribution of stimulus salience, the *Salience Attribution* model is of primary importance for this work. In the main paper, we instigate that brain regions central to the cognitive process of *Salience Attribution* to a distractor should satisfy three conditions, which we shall explain in more detail here:

1. Regions for *Salience Attribution* should be able to separate negative from neutral distractors. Our theoretical framework of attention capture proclaims that distractor images that are rated negative with respect to their emotional content by a general population are more distracting than neutral images. The emotional category ('negative' versus 'neutral') is regarded crucial for such increased distractibility of particular stimuli. The prolonged RTs for negative distractors in our ACES task (see Table 1) are the behavioral evidence for these assumptions. Therefore, we can conclude that any region that cannot distinguish between these categories of stimuli (i.e., emotional content) cannot be central to the salience attribution process. Notably, additional regions may cause distraction (i.e. delayed responses) unrelated to emotional content. However, such regions or alternative sources of salience are not the focus of this study as our operational definition of salience is restricted to the emotional domain.

   In principle, brain regions eligible for *Salience Attribution* may show stronger or weaker fMRI signals for negative as opposed to neutral stimuli. Regions of the 'negative < neutral' fMRI contrast would be expected to show a negative correlation with RTs, because less signal for negative stimuli would indicate longer RTs. However, we are restricting our analysis here to the 'negative > neutral' brain network, because our previous study (Marxen, Jacob, Hellrung, Riedel, & Smolka, 2021) did not show any brain regions that were negatively correlated with RT. In fact, we confirmed this finding in the current data and found no early *Salience Attribution* regions within the negative < neutral fMRI contrast. Thus, it appears that the salience attribution process in the brain is dominated by increased activity for stronger distractor stimuli. In turn, stronger activation for negative distractors is in line with a model that such stimuli recruit more processing resources, which delay the execution of other tasks.

2. Regions for *Salience Attribution* should carry information about the RT within a trial, which is our behavioural measure of distractor salience. We emphasized in our previous work (Marxen et al., 2021) that, while it may be plausible that brain regions such as the amygdala that show differential fMRI (and electrophysiological) signals for negative versus neutral stimuli are responsible for the RT distractor effect that is observed in group studies, the actual evidence for such a claim is weak as long as no trial-by-trial correlations between brain activity and behavior can be observed. Neuroscientific models of attentional capture remain speculative, because it is entirely possible that certain brain regions may be exquisitely sensitive to differences in particular image features (such as features related to emotional valence), but are not pivotal for the attribution of salience and recruitment of cognitive processing resources. However, collecting evidence of trial-by-trial correlations between brain activity and behavioral measures such as RTs can substantiate any claim that brain regions are pivotal for

salience attribution. Such a trial-by-trial correlation is implemented in our model by a neural event regressor with an amplitude that is proportional to the trial-specific RT deviation from the mean RT across the entire run. This parametric modulator GLM approach has been established in fMRI long ago (Buchel, Holmes, Rees, & Friston, 1998) and has been employed in our last paper (Marxen et al., 2021). However, even trial-by-trial correlations with RT are not yet sufficient to prove the causal role of a particular region in mediating the distractor effect. This is due to the fact that RTs in cued tasks are generally variable and that regions that track RT may be related to the execution of the motor response rather than to the processing of the distractor. Thus, a further requirement for causality is temporal precedence of the causal event over the caused event.

3. Regions for *Salience Attribution* should respond to the stimuli early in the processing chain, as they need to influence the subsequent distribution of cognitive resources. This is where CTMC offers a major advance over the existing fMRI analysis methods. Note that the whole cognitive process occurs in approximately 500ms, which is short in comparison to the width of the HRF and the sampling interval of TR = 0.756s. Thus, neural events are modeled (Fig. 1) as stick functions here as opposed to rectangular functions. 'Early' in this context becomes synonymous with time-locked to the onset of the distractor stimulus rather than to the motor response because of the principle nature of the task. This principle nature involves a cued RT task with an additional early causal effector, such as emotional image valence, that influences the RT. Such a task design is one of the major tools of experimental cognitive psychology. Importantly for CTMC, RTs vary on a time scale (std ~= 70ms) that cause a sufficiently variable shift (jitter) in the BOLD fMRI responses to differentiate signals that are time-locked to the effector onset, i.e. the beginning of the cognitive process, from those time-locked to the motor reaction, that marks the end of the cognitive process. Our data show that such a temporal differentiation is not only replicable but also largely in line with existing knowledge on brain organization (see below). Importantly, this differentiation is not possible in the conventional fMRI GLM framework. Adding an additional motor regressor into the GLM model on top of the distractor onset regressor is not feasible, because the vascular response is broad (~5s) and would introduce strong co-linearity between regressors that are separated by hundreds of ms only, which would in turn render fitting the data unstable. In addition, GLM models may be extended by the temporal derivative of a condition in order to measure the latency of the hemodynamic response (Calhoun, Stevens, Pearlson, & Kiehl, 2004). We actually considered such temporal derivatives for all models to investigate whether these would improve the models and found that this was not the case for most of the brain (see Fig. S4). 85% of all brain voxels are assigned the same model or in the not-replicable category in both comparisons. This observation indicates that model selection between early and late models was not driven by the mean delay of the response with respect to the distractor onset (~[400+200]ms, see Fig. 1) but rather by the variability of the RT. This observation is crucial, because knowledge of the hemodynamic latency with respect to the distractor onset would not have allowed us to draw conclusions about relative neural latencies, as discussed in the main paper. Our results show that model comparison (CTMC), in contrast to a GLM model with additional regressors, does have the necessary sensitivity to separate a cognitive process into an early and a late phase. Thus,

CTMC increased the temporal resolution of fMRI to time scales relevant for cognitive processes without being vulnerable to misinterpretation due to hemodynamic delays. The insula region is particularly sensitive to such misinterpretations because data by Chang et al. ((Chang, Thomason, & Glover, 2008) and personal communications) indicate shorter latencies of the hemodynamic response in the insula compared to most of the cortex.

This temporal resolution of CTMC is not yet on par with electrophysiological measures that are capable to resolve timing difference on the order of neural conduction times between directly connected neurons (~10ms, (Carretie, Yadav, & Mendez-Bertolo, 2021)). However, it is not obvious that such a high resolution is required to identify the role of local neuronal ensembles (as measured by all non-invasive neuroimaging techniques) because such ensembles remain active for hundreds of milliseconds, are interconnected locally, and receive additional inputs from other regions that may be crucial for subsequent cognitive processes such as the recruitment of attention (Gothard, 2020). Such interactions would require additional time. The question, for example, when exactly the brain is capable to distinguish emotional from non-emotional signals (Carretie et al., 2021) may not be very helpful to understand the neurocognitive process of attention capture for two reasons. First of all, the information on valence is already there when an image is projected onto the retina, i.e. this information could theoretically be extracted by a measurement device. Secondly, the pure classification of a stimulus may not yet be synonymous with the attribution of salience as it is conceivable that the same stimulus may be more or less salient, i.e. distracting, dependent on other factors like the momentary brain state of the subject as the stimulus is perceived. One brain state may lead to the recruitment of attention and cognitive resources, another not. Thus, it is not sufficient for a *Salience Attribution* region to merely differentiate negative from neutral images, but the prediction of RTs is crucial.

The association of a particular cognitive process with a specific fMRI model is often not trivial. The yellow cluster in the visual system in Fig. 2, for example, indicates a late activation that we assigned to the *Task Execution* process. But it is not clear whether this association is really appropriate given that the signal may, for example, also be related to a deeper processing of the distractors images rather than to an evaluation of the task cues. In general, we cannot observe cognitive processes directly but only infer them from observations. CTMC allows us to test related hypotheses that include process timing; but, the interpretation of the data in terms of cognitive processes remains challenging and will still require converging evidence from multiple experimental manipulations. Additionally, we cannot rule out the possibility that certain brain areas (e.g., the amygdala) are still involved in certain cognitive processes (i.e., *Salience Attribution*), even though the fMRI signal does not indicate this.

Supplementary Results

Behavioral results are summarized in Table 1 for RTs and Table S1 for error rates for Study A, Study B1 and Study B2. Further details of the fMRI model comparison are provided in Fig. S1-S5. Fig. S1 is a more detailed version of Fig. 2 of the main manuscript and will be explained in more detail below. Fig. S2 is a mosaic version of the replicably-winning-model map used in Fig. 2 and Fig. S1 and provides additional sagittal views of the model

comparison results within the negative > neutral distractor ROI. Fig. S3 is a whole brain version of this replicably-winning-model map. Figs. S4 and S5 are comparing the whole-brain replicably-winning-model map from Fig. S3 with an equivalent map for an 8-model comparison, which included the original four models from the main paper plus four additional models with temporal derivatives, which mimic latency shifts of the HRF. Digital versions of the 3D winning model maps, a manual for easy viewing of these maps using MRICroGL (https://www.nitrc.org/projects/mricrogl/), and two (ROI and whole brain) Excel tables, which contain information about each cluster and how much it overlaps with a particular atlas region, are provided in the Suppl. Data S1 at https://osf.io/kqz6a/ as part of the Open Science Foundation project "SFB940/1-A7: Volitional Control of Brain Activity: Effects of Neurofeedback on Emotional Reactivity" (https://osf.io/6afq5/). Fig. S6 and Table S2 show the results of the replication of Study A in Study B1.

Explanation of Fig. S1

While the ACES task served in the main manuscript primarily as an application example of the CMTC method, the cognitive process of attention capture is a very important human capacity and intensely studied by experimental psychologists and cognitive neuroscientists. Thus, we will discuss our results and their implications in more detail here, going beyond the amygdala and the anterior insula already explained in the main manuscript. We will still focus on regions that are part of the 'negative > neutral' distractor contrast (see Fig. S1).

First, we would like to focus on the early visual pathway. It is well known that visual information is passed from the eye via the optic nerve to the 'visual' thalamus, and subsequently via the optic radiations to the primary visual cortex (cluster 25) (Carretie et al., 2021). In their latest review, Carretie et al. (Carretie et al., 2021) emphasize the potential role of the visual thalamus, specifically the lateral geniculate nucleus (LGN), the pulvinar and the thalamic reticular nucleus (TRN), as initial evaluation structures (IES). The authors argue that cortical regions such as the amygdala, insula and ventral prefrontal Cortex (vPFC) are responding too slowly to explain early electrophysiological response differences between valence classes in the visual cortex at 80ms. Amygdala, insula and vPFC show such differences at the earliest at 74ms, which would be too late to transmit the information to the visual cortex in time. LGN responses, in turn, show response differences already at 20-40ms. Consistent with this prior knowledge, the thalamus (cluster 15) displays the features of an early *Distractor Perception* region in our current analysis. However, our operational definition of salience attribution requires a measurable prediction on behaviour (i.e., RTs). In this regard, we find no evidence in our winning-model map that such attribution already occurs in the thalamus. In fact, neither the primary visual cortex (cluster 25) nor secondary visual/attention regions (clusters 50 and 5) show such predictive powers. It is conceivable that the necessary amplification of emotional stimuli to cause distraction with measurable behavioral differences requires the involvement of higher order cortical regions such as the dAI.

Second, the replicably-winning-model map associates particular subregions of the negative > neutral contrast ROI with one of the four fMRI models (see Data S1 for a complete list). Regions that show *Distractor Perception* properties include the dorsal attention network regions fundus of the superior temporal gyrus (FST) and the inferotemporal areas PH and TE2 (cluster 5) as well as the frontal and premotor eye fields (cluster 13), the LB amygdala

(cluster 14), the salience network regions anterior insula and area 55b (cluster 13) and default mode network regions close to the insula region (cluster 13) and in the orbito-frontal cortex (area 10v, cluster 10).

Within the ROI, only the salience network regions dAI (cluster 41) and SMA (cluster 48) and the control network region sIFG (area 6, cluster 47) are predictive of RT, i.e. considered early *Salience Attribution* regions. Importantly, other regions modulated by RT such as the default mode network region vAI (cluster 97) and the control network region 8BM (cluster 106) are only later activated Modulated *Task Execution* regions. Note that the SMA region (cluster 48) actually converts largely from the *Salience Attribution* model to the *Modulated Task Execution* + Derivates model when temporal derivatives are additionally included (see Suppl. Data S1 and Fig. S4&5). Thus, only dAI and sIFG remain as the core salience attribution regions. In addition to the dAI as a pivotal salience attribution region, it is highly interesting that the sIFG also shows *Salience Attribution* properties. This region is rarely considered in this context and deserves more attention in future investigations.

A number of regions close to Early Perception regions are closer related with the motor response, i.e. considered later activating *Task Execution* regions, such as the visual network region FFC (cluster 50), the dorsal attention network area TF (cluster 50), the limbic area 10v (cluster 56), the default mode area 9 (cluster 56) and the SF amygdala (cluster 61). The spreadsheet file cluster_stats_atlas_ROIs_neg_larger_neu_dense.xlsx of the Suppl. Data S1 (https://osf.io/kqz6a/ -> subdirectory \MRICroGL_Files\Winning_Model_Maps\) contains a detailed list of atlas regions for each cluster of the winning-model map.

Third, we would like to elaborate on the results for the amygdala and insula, which are considered key regions for attentional capture by emotional stimuli. In addition to confirming the anterior insula as a salience attribution region, the observed functional segregation of the anterior insula signal in Fig. 3 is in line with insula cytoarchitectonics (Augustine, 1996; Mesulam & Mufson, 1982). It is also consistent with fMRI studies linking vAI and dAI to distinct networks and functions, respectively. For example, Deen et al. (Deen, Pitskel, & Pelphrey, 2011) found that the vAI was closely linked with pregenual ACC (pACC) and the default mode network (DMN). Neural activity in dAI, on the other hand, was primarily correlated with the dorsal anterior cingulate cortex (dACC). Notably, dAI and dACC anchor the salience network (SN) and relate to the cognitive control network (for a review see also (Uddin, 2015)). Interestingly, examination of the insula using yet another approach, namely quantitative modelling of multi-shell diffusion MRI data, yields the same organizational differentiation of this region (Menon et al., 2020). Thus, the CTMC results presented here are clearly consistent with previous parcellations of the insula into dorsal and ventral subregions. Furthermore, our results match with prior knowledge on the functional relevance of the insula in salience processing and extend this knowledge to include the temporal sequence of events.

For the amygdala, our finding (see Fig. 4) were less anticipated from the existing literature; they rather complement existing knowledge on amygdala subdivision. First of all, it is to be noted that the negative > neutral distractor ROI covers only 23% of the left amygdala and 22% of the right amygala and even less of the LB (< 10%), the by far largest subdivision, using the probability maps of the Anatomy Toolbox by Amunts et al. (Amunts et al., 2005) (see Suppl. Data S1). Activation (negative > neutral) is found in all

subdivisions in both hemispheres as might be expected from the well-known emotion-related functions of the amygdala (Davis & Whalen, 2001; Gothard, 2020; Phan, Wager, Taylor, & Liberzon, 2002; Phelps & LeDoux, 2005) and also by the view that the amygdala serves as a multi-dimensional classifier potentially without strict categorical subdivisions (Gothard, 2020). Within all subdivisions, we confirmed our previous results (Marxen et al., 2021) that the amygdala signal is not associated with RT; only *Distractor Perception* and *Task Execution* components were found. Interestingly, the *Distractor Perception* components dominated the activity in the right latero-basal (LB) amygdala (8 of 10 voxels) only. All other subdivisions of the amygdala were dominated by the later *Task Execution* component. Past studies have consistently found distinct function and functional connectivity in these sub-regions (Bzdok, Laird, Zilles, Fox, & Eickhoff, 2013; Caparelli et al., 2017; Kim et al., 2011; Roy et al., 2009). Hemispheric lateralization in amygdala function is also known (Baas, Aleman, & Kahn, 2004; Fruhholz et al., 2015; Gainotti, 2020; Ji & Neugebauer, 2009; Palomero-Gallagher & Amunts, 2021; Zhang et al., 2018). Our finding supplements the previous notion that the LB is primarily associated with significance detection in high-level sensory input, CM with mediating attentional and motor responses as a main output centre, and SF with olfactory and social information processing (Bzdok et al., 2013). The findings suggest that LB, specifically in the right hemisphere, is dominated by inputs from primary visual regions (such as the visual thalamus). Also note that amygdala parcellations vary depending on the data used [for example: cytoarchitectonics (Amunts et al., 2005), functional connectivity (Roy et al., 2009), structural connectivity (Bach, Behrens, Garrido, Weiskopf, & Dolan, 2011)] or the methods used [for example: recurrence quantification analysis (Bielski, Adamus, Kolada, Raczaszek-Leonardi, & Szatkowska, 2021), plausibility guided hierarchical clustering (Klein-Flügge, 2020), semi-supervised spectral clustering (Zhang et al., 2018)]. This variation may explain the low overlap of Amunts' LB amygdala ROI with the Neg > Neu contrast in our data, although a stronger involvement of the LB nuclei might be expected.

In summary, Fig. 2 and S1 demonstrate that CTMC results are congruent with numerous previous findings on brain functions and parcellations and offer additional insights related to the timing of cognitive processes.

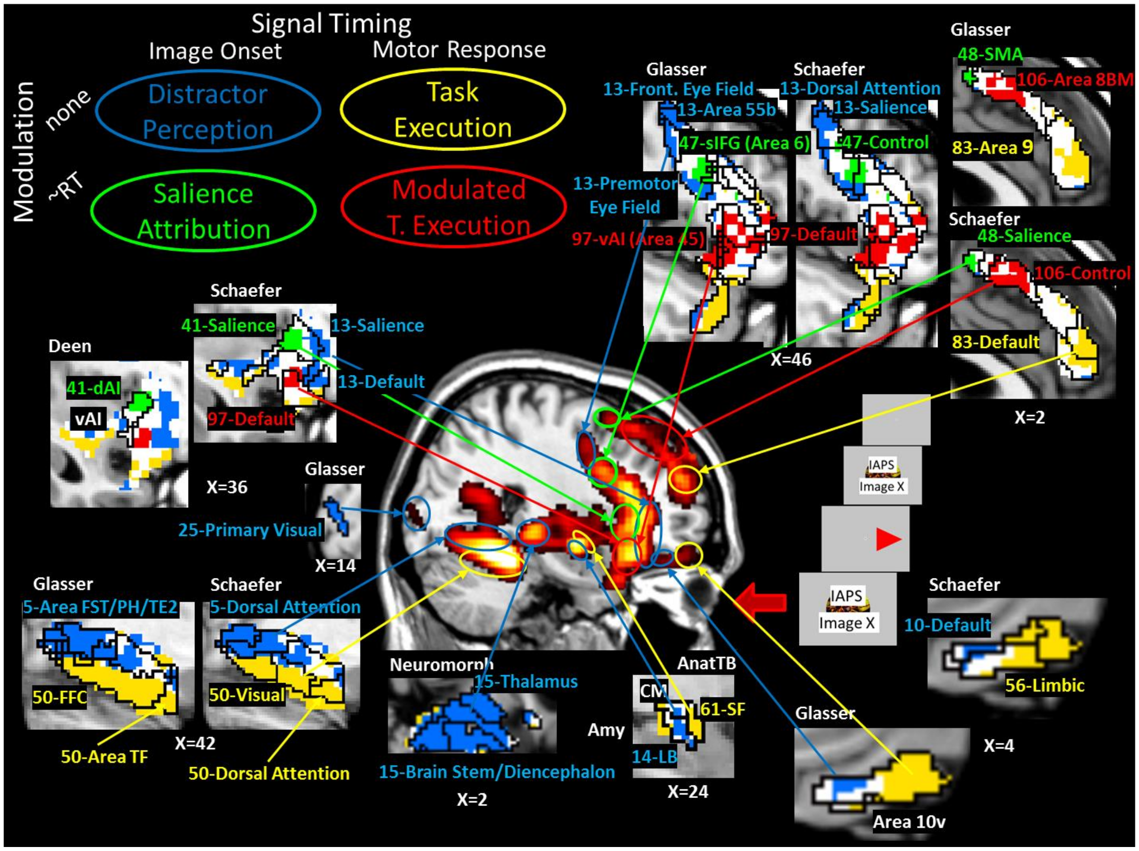

Fig. S1. Detailed version of Fig. 2 of the main manuscript. This figure illustrates core brain regions that are known to be associated with attention capture by visual emotional stimuli (ACES). The central heat map image is a maximum intensity projection through the right hemisphere of the fMRI signal difference negative > neutral distractors ($P < 0.001$ uncor. group data for N=40 from Study A) overlaid on a sagittal slice of a T1-weighted structural brain scan. For core regions, cut-outs of a single slice at the given sagittal slice position X in MNI space are shown with replicably-winning-model maps overlaid. The different colors represent the winning model over all participants within each region as indicated in the upper left. White regions were not replicable. Regional boundaries are drawn based on different brain atlases (see Suppl. Methods) as indicated above each cut-out. Winning model clusters are labeled with their cluster number and the respective atlas label (functional network). This figure is further explained in the Suppl. Text above. (Amy – amygdala, CM – centromedial, SF – superficial, LB – laterobasal subregions, dAI - dorsal anterior insula, vAI - ventral anterior insula, FFC – fusiform face complex, RT – reaction time, SMA – supplementary motor area).

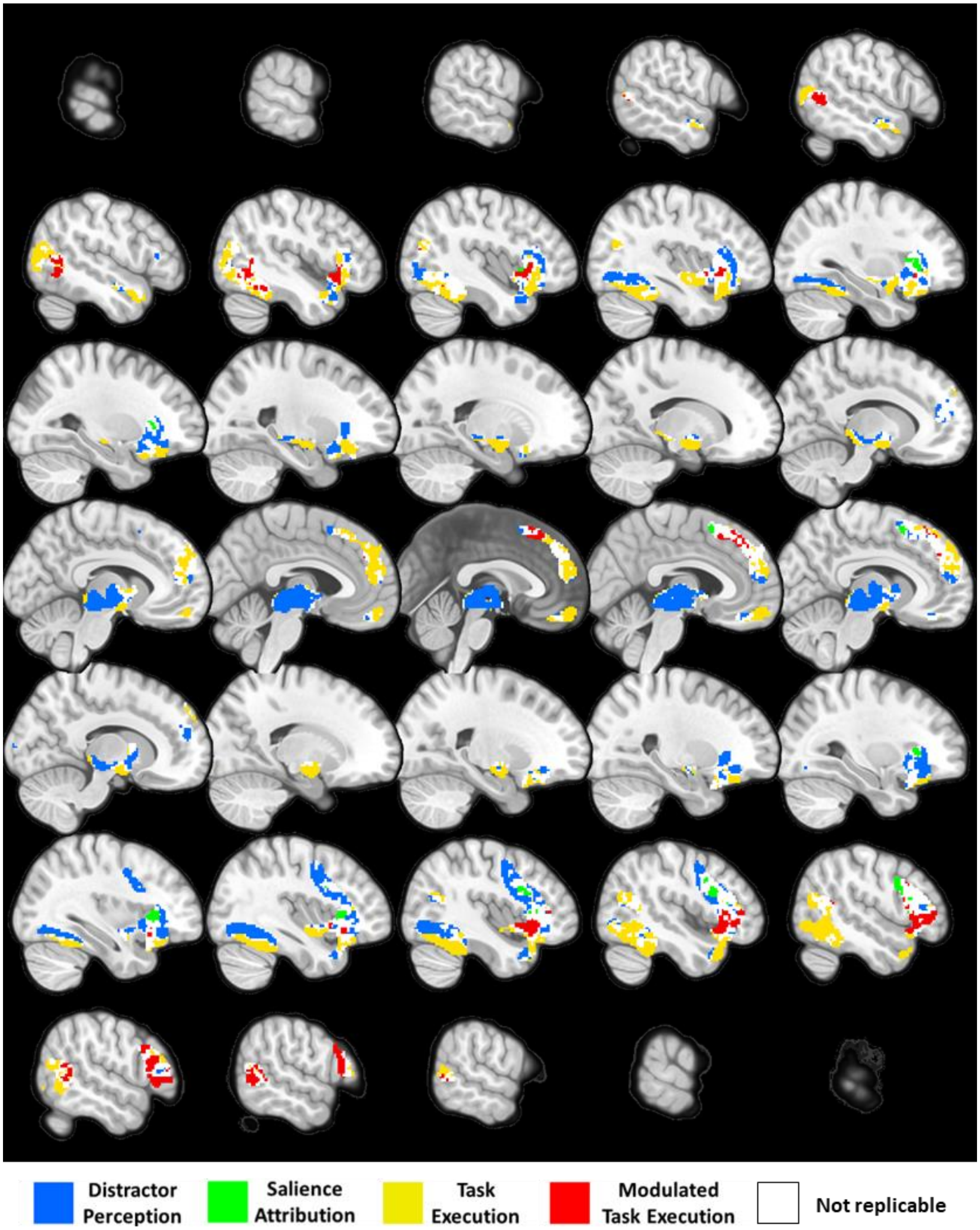

Fig. S2. Mosaic display of the replicably-winning-model map used in Fig. 2 and S1 for sagittal slices for X = -70 to +72 mm in MNI space. In Study B, the four models explained in Fig. 1 were compared within the negative > neutral distractor ROI from Study A. White regions were not replicated between Study B1 (N=40) and B2 (N=33). 65% of all voxels within the ROI were replicated.

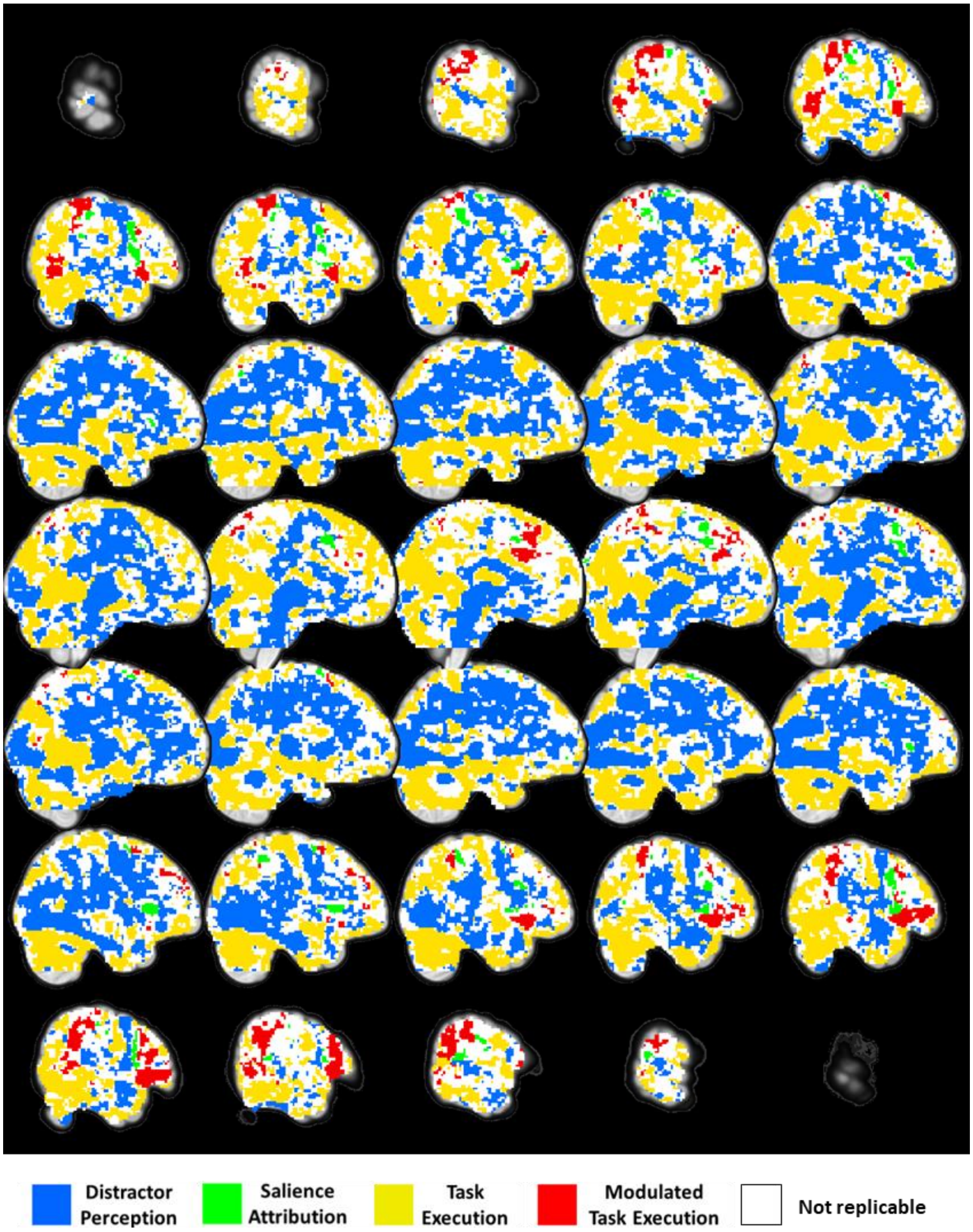

Fig. S3. Mosaic display of replicably-winning-model map equivalent to Fig. S2 but for the whole-brain mask. 64% of all voxels within the whole-brain mask were replicated.

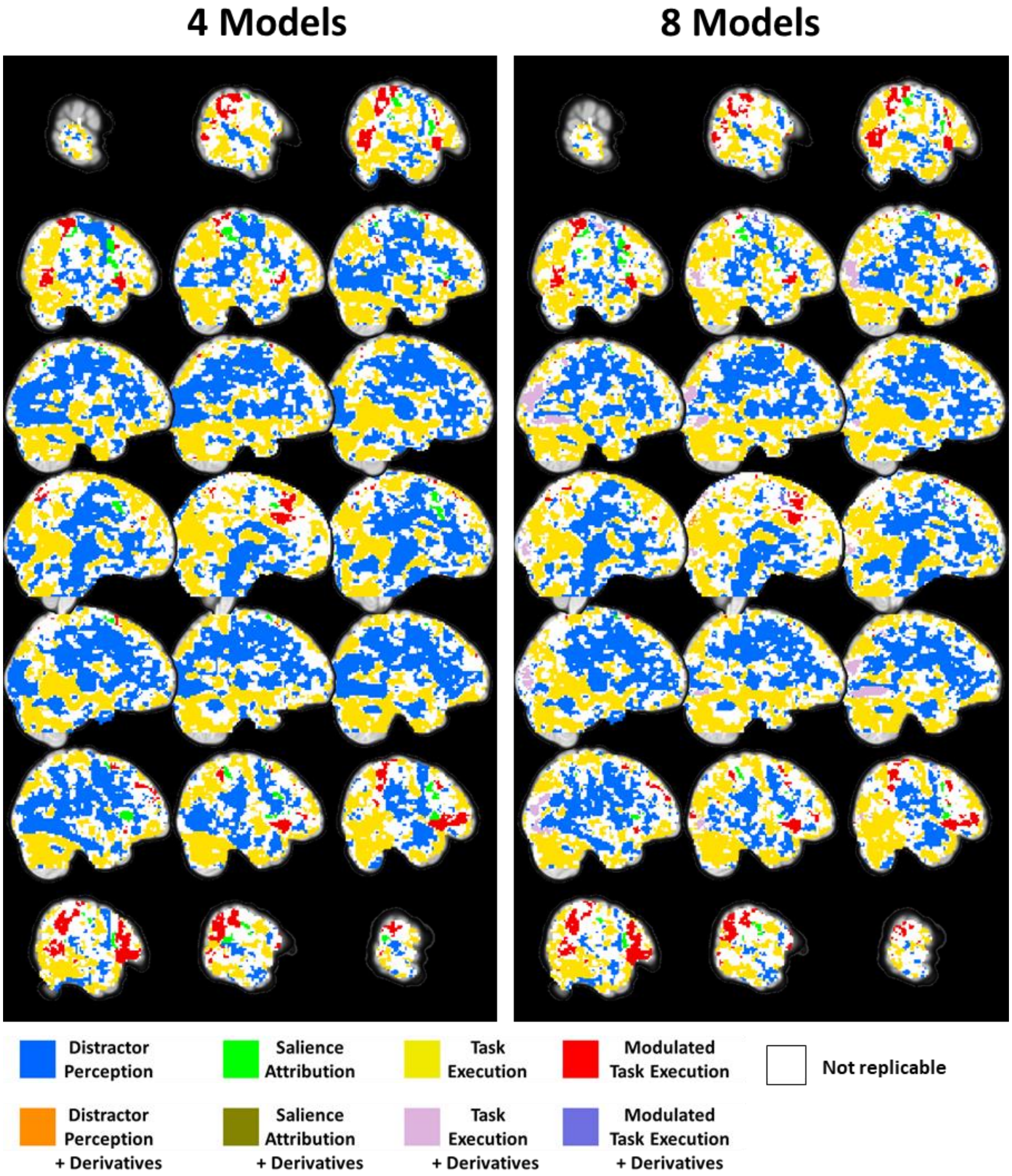

Fig. S4. Comparison of replicably-winning-model maps (MNI X = -68 to +68 mm) for the 4-model comparison as in Fig. S3 (left) and the 8-model comparison that includes temporal derivatives (right). 85% of all brain voxels are assigned the same model (or not-replicable). Replicability for the 8-model comparison on the right is 61% as compared to 64% on the left. Models with derivatives are winning replicably in less than 2% of all brain voxels.

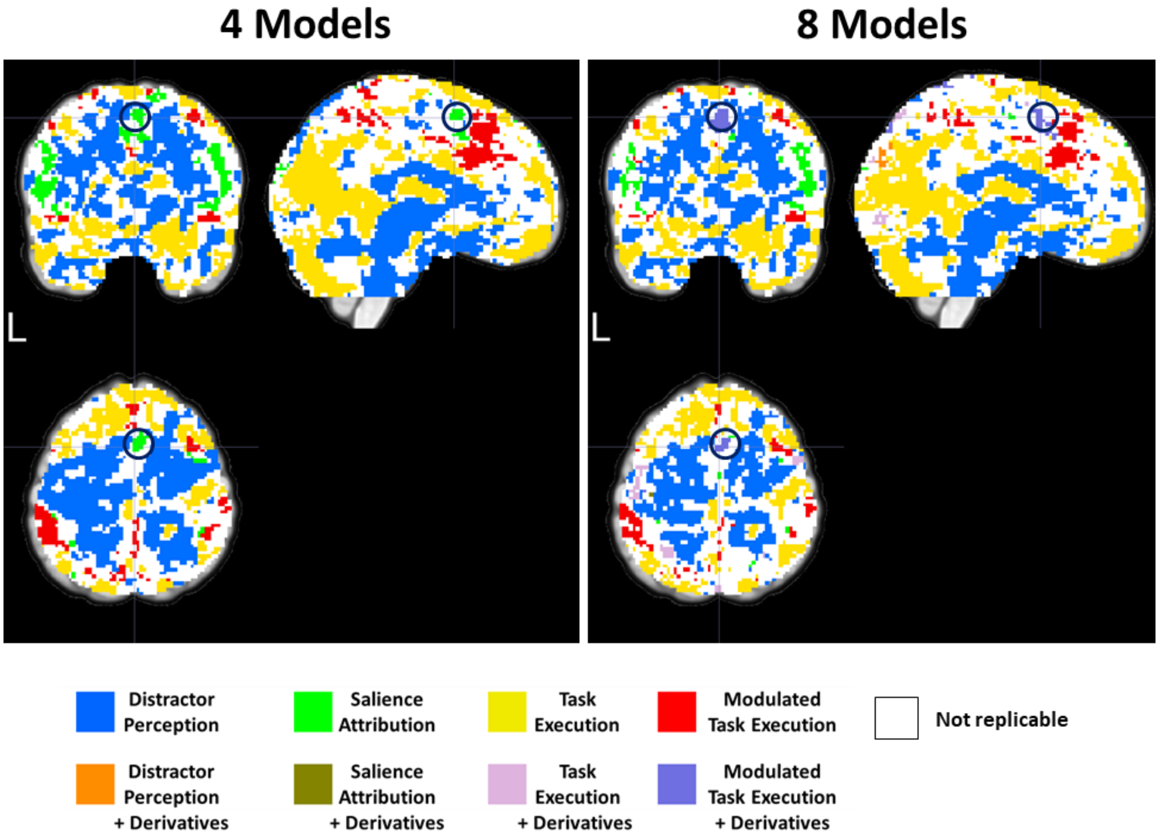

Fig. S5. Comparison of replicably-winning-model maps for the 4-model comparison (left) and the 8-model comparison that includes temporal derivatives (right); same as Fig. S4 but focusing on the SMA region (black circle).

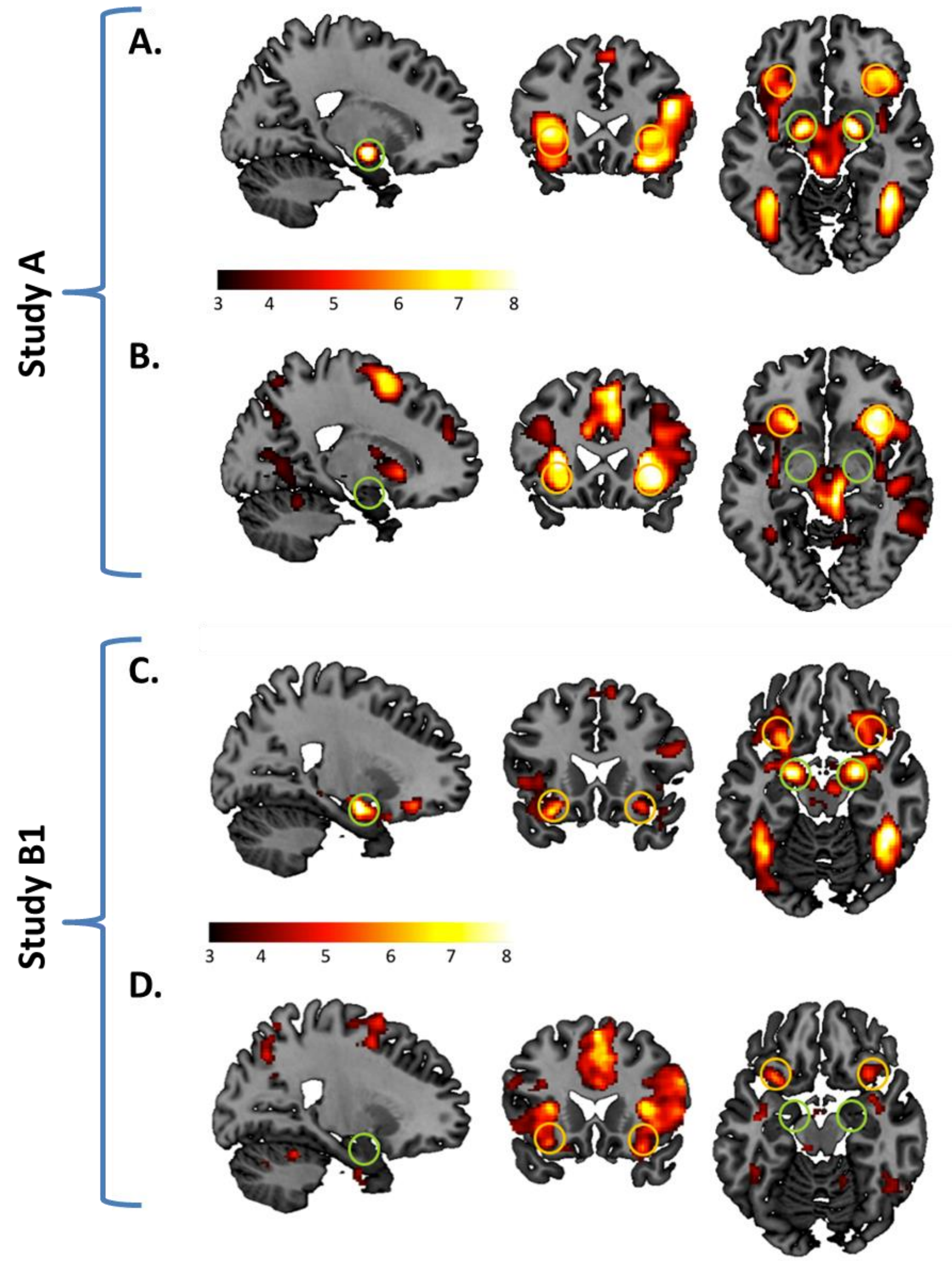

Fig. S6. Replication of Neuroimaging Results from Study A in Study B. Shown are group activation maps for the Valence (A. & C.) and RT Model (B. & D.) as described in (1) for data acquired in Study A (A. & B.) and Study B1 (C. & D.). Group activation maps (N=40, puncorr < 0.001) are shown for the contrast „Negative > Neutral Distractor Images“ of the Valence Model (T117 > 3.16) (A. & C.) and the parametric contrast „Positive Correlation with RT“ for the RT Model (T39 > 3.31) (B. & D.). The RT Model is equivalent to the *Salience Attribution* Model in the current study. Green circles mark the amygdala, yellow circles the anterior insula.

Table S1. Error Rates for Negative and Neutral Distractors. Group mean error rates are presented with standard error (SE) together with results of one-sided paired t tests, the effect sizes Cohen's d and dz as with 95% confidence intervals (CIs). T-values are not significant ('Neg' – negative, 'Neu' – neutral).

		Error rates % (SE)		Neg vs. Neu			
	N	Neg	Neu	Neg-Neu	*t*	*Cohens' d (95% CI)*	d_z (95% CI)
Study A	44	1.50 ± 0.36	1.03 ± 0.29	0.47 ± 0.31	1.15	0.22 (-0.14 – 0.82)	0.17 (-0.14 – 0.46)
Study B1	40	1.96 ± 0.39	1.94 ± 0.43	0.02 ± 0.36	0.06	0.01 (-0.22 - 0.38)	0.01 (-0.34 - 0.33)
Study B2	35	1.88 ± 0.38	1.64 ± 0.46	0.23 ± 0.38	0.38	0.09 (-0.30 - 0.61)	0.06 (-0.27 - 0.41)

Table S2. Group Activation Results for the (I) Valence and (II) RT Model for Study B1 (see Fig. S6 C & D). A cluster threshold of $p < 0.001$ and a small volume correction with pFWE < 0.05 using a bilateral amygdala and bilateral anterior insula mask was applied to (I) the contrast „Negative > Neutral Distractor Images" of the Valence Model and (II) the parametric contrast „Positive Correlation with RT" for the RT Model. Clusters covering part of the amygdala are marked with "*". This table is equivalent to Tab. 2 in (1). (Amy – amygdala, WM – white matter, PHG - parahippocampal gyrus, AI – anterior insula, Ent - entorhinal area).

Cluster-Level			Peak-Level			MNI coordinate [mm]			
$P_{FWE-corr}$	k_E	P_{uncorr}	$P_{FWE-corr}$	T	$P_{un-corr.}$	x	y	z	Anatomical Region
			I) Group Contrast: Negative > Neutral						
0.000	272	0.000	0.000	8.69	0.000	22	-4	-16	R Amy
0.000	298	0.000	0.000	8.46	0.000	-20	-6	-16	L Amy
			0.000	6.17	0.000	-28	2	-28	L Cerebral WM*
			0.044	3.96	0.000	-30	-8	-32	L PHG*
0.002	114	0.005	0.000	6.81	0.000	-26	14	-18	L AI
0.007	78	0.016	0.000	5.22	0.000	30	18	-14	R AI
			0.011	4.40	0.000	36	12	-4	
0.129	12	0.307	0.009	4.45	0.000	32	-4	-36	R Cerebral WM*
			0.021	4.19	0.000	34	-6	-32	R Cerebral WM*
0.048	31	0.108	0.038	4.01	0.000	-34	10	-2	L AI
			0.043	3.97	0.000	-38	8	-6	
0.271	2	0.699	0.040	4.00	0.000	28	4	-30	R Ent*
			II) Group Contrast: Positive Correlation with RT						
0.000	571	0.000	0.000	7.42	0.000	-30	22	8	L AI
			0.000	6.94	0.000	-32	24	-4	
			0.000	6.51	0.000	-38	16	-8	
			0.000	6.21	0.000	-30	16	-14	
			0.002	5.42	0.000	-36	10	2	
			0.003	5.35	0.000	-26	14	-18	
			0.006	5.08	0.000	-36	0	10	
			0.006	5.05	0.000	-38	2	-4	
0.000	569	0.000	0.000	7.32	0.000	34	16	6	R AI
			0.000	6.06	0.000	42	-2	4	
			0.001	5.78	0.000	34	24	-8	
			0.001	5.69	0.000	38	6	2	
			0.001	5.61	0.000	40	20	-4	
			0.002	5.48	0.000	38	10	-8	
			0.005	5.13	0.000	32	18	-16	

SI References

Amunts, K., Kedo, O., Kindler, M., Pieperhoff, P., Mohlberg, H., Shah, N. J., . . . Zilles, K. (2005). Cytoarchitectonic mapping of the human amygdala, hippocampal region and entorhinal cortex: intersubject variability and probability maps. *Anat Embryol (Berl), 210*(5-6), 343-352. doi:10.1007/s00429-005-0025-5

Augustine, J. R. (1996). Circuitry and functional aspects of the insular lobe in primates including humans. *Brain Res Brain Res Rev, 22*(3), 229-244. doi:10.1016/s0165-0173(96)00011-2

Baas, D., Aleman, A., & Kahn, R. S. (2004). Lateralization of amygdala activation: a systematic review of functional neuroimaging studies. *Brain Res Brain Res Rev, 45*(2), 96-103. doi:10.1016/j.brainresrev.2004.02.004

Bach, D. R., Behrens, T. E., Garrido, L., Weiskopf, N., & Dolan, R. J. (2011). Deep and superficial amygdala nuclei projections revealed in vivo by probabilistic tractography. *J Neurosci, 31*(2), 618-623. doi:10.1523/JNEUROSCI.2744-10.2011

Bielski, K., Adamus, S., Kolada, E., Raczaszek-Leonardi, J., & Szatkowska, I. (2021). Parcellation of the human amygdala using recurrence quantification analysis. *Neuroimage, 227*, 117644. doi:10.1016/j.neuroimage.2020.117644

Buchel, C., Holmes, A. P., Rees, G., & Friston, K. J. (1998). Characterizing stimulus-response functions using nonlinear regressors in parametric fMRI experiments. *Neuroimage, 8*(2), 140-148. doi:10.1006/nimg.1998.0351

Bzdok, D., Laird, A. R., Zilles, K., Fox, P. T., & Eickhoff, S. B. (2013). An investigation of the structural, connectional, and functional subspecialization in the human amygdala. *Hum Brain Mapp, 34*(12), 3247-3266. doi:10.1002/hbm.22138

Calhoun, V. D., Stevens, M. C., Pearlson, G. D., & Kiehl, K. A. (2004). fMRI analysis with the general linear model: removal of latency-induced amplitude bias by incorporation of hemodynamic derivative terms. *Neuroimage, 22*(1), 252-257. doi:10.1016/j.neuroimage.2003.12.029

Caparelli, E. C., Ross, T. J., Gu, H., Liang, X., Stein, E. A., & Yang, Y. (2017). Graph theory reveals amygdala modules consistent with its anatomical subdivisions. *Sci Rep, 7*(1), 14392. doi:10.1038/s41598-017-14613-4

Carretie, L., Yadav, R. K., & Mendez-Bertolo, C. (2021). The Missing Link in Early Emotional Processing. *Emotion Review, 13*(3), 225-244. doi:10.1177/17540739211022821

Chang, C., Thomason, M. E., & Glover, G. H. (2008). Mapping and correction of vascular hemodynamic latency in the BOLD signal. *Neuroimage, 43*(1), 90-102. doi:10.1016/j.neuroimage.2008.06.030

Davis, M., & Whalen, P. J. (2001). The amygdala: vigilance and emotion. *Mol Psychiatry, 6*(1), 13-34. doi:10.1038/sj.mp.4000812

Deen, B., Pitskel, N. B., & Pelphrey, K. A. (2011). Three systems of insular functional connectivity identified with cluster analysis. *Cereb Cortex, 21*(7), 1498-1506. doi:10.1093/cercor/bhq186

Fruhholz, S., Hofstetter, C., Cristinzio, C., Saj, A., Seeck, M., Vuilleumier, P., & Grandjean, D. (2015). Asymmetrical effects of unilateral right or left amygdala damage on auditory cortical processing of vocal emotions. *Proc Natl Acad Sci U S A, 112*(5), 1583-1588. doi:10.1073/pnas.1411315112

Gainotti, G. (2020). *Emotions and the Right Side of the Brain*: Springer.

Gothard, K. M. (2020). Multidimensional processing in the amygdala. *Nat Rev Neurosci, 21*(10), 565-575. doi:10.1038/s41583-020-0350-y

Ji, G., & Neugebauer, V. (2009). Hemispheric lateralization of pain processing by amygdala neurons. *J Neurophysiol, 102*(4), 2253-2264. doi:10.1152/jn.00166.2009

Kim, M. J., Loucks, R. A., Palmer, A. L., Brown, A. C., Solomon, K. M., Marchante, A. N., & Whalen, P. J. (2011). The structural and functional connectivity of the amygdala: from normal emotion to pathological anxiety. *Behav Brain Res, 223*(2), 403-410. doi:10.1016/j.bbr.2011.04.025

Klein-Flügge, M. C. J., D.E.A.; Takagi, Y.; Verhagen, L.; Smith, S.M.; Rushworth M.F.S. (2020). Anatomically precise relationship between specific amygdala connections and selective markers of mental well-being in humans. *bioRxiv*. doi:10.1101/2020.03.08.980995

Marxen, M., Jacob, M. J., Hellrung, L., Riedel, P., & Smolka, M. N. (2021). Questioning the role of amygdala and insula in an attentional capture by emotional stimuli task. *Hum Brain Mapp, 42*(5), 1257-1267. doi:10.1002/hbm.25290

Menon, V., Gallardo, G., Pinsk, M. A., Nguyen, V. D., Li, J. R., Cai, W., & Wassermann, D. (2020). Microstructural organization of human insula is linked to its macrofunctional circuitry and predicts cognitive control. *Elife, 9*. doi:10.7554/eLife.53470

Mesulam, M. M., & Mufson, E. J. (1982). Insula of the old world monkey. I. Architectonics in the insulo-orbito-temporal component of the paralimbic brain. *J Comp Neurol, 212*(1), 1-22. doi:10.1002/cne.902120102

Palomero-Gallagher, N., & Amunts, K. (2021). A short review on emotion processing: a lateralized network of neuronal networks. *Brain Struct Funct*. doi:10.1007/s00429-021-02331-7

Phan, K. L., Wager, T., Taylor, S. F., & Liberzon, I. (2002). Functional neuroanatomy of emotion: a meta-analysis of emotion activation studies in PET and fMRI. *Neuroimage, 16*(2), 331-348. doi:10.1006/nimg.2002.1087

Phelps, E. A., & LeDoux, J. E. (2005). Contributions of the amygdala to emotion processing: from animal models to human behavior. *Neuron, 48*(2), 175-187. doi:10.1016/j.neuron.2005.09.025

Roy, A. K., Shehzad, Z., Margulies, D. S., Kelly, A. M., Uddin, L. Q., Gotimer, K., . . . Milham, M. P. (2009). Functional connectivity of the human amygdala using resting state fMRI. *Neuroimage, 45*(2), 614-626. doi:10.1016/j.neuroimage.2008.11.030

Uddin, L. Q. (2015). Salience processing and insular cortical function and dysfunction. *Nat Rev Neurosci, 16*(1), 55-61. doi:10.1038/nrn3857

Zhang, X., Cheng, H., Zuo, Z., Zhou, K., Cong, F., Wang, B., . . . Fan, Y. (2018). Individualized Functional Parcellation of the Human Amygdala Using a Semi-

supervised Clustering Method: A 7T Resting State fMRI Study. *Front Neurosci, 12*, 270. doi:10.3389/fnins.2018.00270
